# Facilitation of colonic T cell immune responses is associated with an exacerbation of dextran sodium sulfate–induced colitis in mice lacking microsomal prostaglandin E synthase-1

**DOI:** 10.1186/s41232-021-00188-1

**Published:** 2022-01-04

**Authors:** Fumiaki Kojima, Hiroki Sekiya, Yuka Hioki, Hitoshi Kashiwagi, Makoto Kubo, Masaki Nakamura, Shotaro Maehana, Yoshitaka Imamichi, Koh-ichi Yuhki, Fumitaka Ushikubi, Hidero Kitasato, Takafumi Ichikawa

**Affiliations:** 1 Department of Pharmacology, Kitasato University School of Allied Health Sciences, 1-15-1 Kitasato, Sagamihara, 252-0373 Japan; 2 Department of Regulation Biochemistry, Kitasato University Graduate School of Medical Sciences, 1-15-1 Kitasato, Sagamihara, 252-0373 Japan; 3 Regenerative Medicine and Cell Design Research Facility, 1-15-1 Kitasato, Sagamihara, 252-0373 Japan; 4 Department of Pharmacology, Asahikawa Medical University, 2-1-1-1 Midorigaoka higashi, Asahikawa, 078-8510 Japan; 5 Division of Clinical Immunology, Graduate School of Medical Sciences, Kitasato University, 1-15-1 Kitasato, Minami-ku, Sagamihara, 252-0373 Japan; 6 Department of Environmental Microbiology, Kitasato University Graduate School of Medical Sciences, 1-15-1 Kitasato, Sagamihara, 252-0373 Japan

**Keywords:** Inflammatory bowel disease, Colitis, Immunity, Th17 and Th1 response, Cytokine, Cyclooxygenase, Prostaglandin E synthase, Prostaglandin E_2_

## Abstract

**Background:**

Microsomal prostaglandin E synthase-1 (mPGES-1) is a key enzyme that acts downstream of cyclooxygenase and plays a major role in inflammation by converting prostaglandin (PG) H_2_ to PGE_2_. The present study investigated the effect of genetic deletion of mPGES-1 on the development of immunologic responses to experimental colitis induced by dextran sodium sulfate (DSS), a well-established model of inflammatory bowel disease (IBD).

**Methods:**

Colitis was induced in mice lacking mPGES-1 (mPGES-1^−/−^ mice) and wild-type (WT) mice by administering DSS for 7 days. Colitis was assessed by body weight loss, diarrhea, fecal bleeding, and histological features. The colonic expression of mPGES-1 was determined by real-time PCR, western blotting, and immunohistochemistry. The impact of mPGES-1 deficiency on T cell immunity was determined by flow cytometry and T cell depletion *in vivo*.

**Results:**

After administration of DSS, mPGES-1^−/−^ mice exhibited more severe weight loss, diarrhea, and fecal bleeding than WT mice. Histological analysis further showed significant exacerbation of colonic inflammation in mPGES-1^−/−^ mice. In WT mice, the colonic expression of mPGES-1 was highly induced on both mRNA and protein levels and colonic PGE_2_ increased significantly after DSS administration. Additionally, mPGES-1 protein was localized in the colonic mucosal epithelium and infiltrated inflammatory cells in underlying connective tissues and the lamina propria. The abnormalities consistent with colitis in mPGES-1^−/−^ mice were associated with higher expression of colonic T-helper (Th)17 and Th1 cytokines, including interleukin 17A and interferon-γ. Furthermore, lack of mPGES-1 increased the numbers of Th17 and Th1 cells in the lamina propria mononuclear cells within the colon, even though the number of suppressive regulatory T cells also increased. CD4^+^ T cell depletion effectively reduced symptoms of colitis as well as colonic expression of Th17 and Th1 cytokines in mPGES-1^−/−^ mice, suggesting the requirement of CD4^+^ T cells in the exacerbation of DSS-induced colitis under mPGES-1 deficiency.

**Conclusions:**

These results demonstrate that mPGES-1 is the main enzyme responsible for colonic PGE_2_ production and deficiency of mPGES-1 facilitates the development of colitis by affecting the development of colonic T cell–mediated immunity. mPGES-1 might therefore impact both the intestinal inflammation and T cell–mediated immunity associated with IBD.

**Supplementary Information:**

The online version contains supplementary material available at 10.1186/s41232-021-00188-1.

## Introduction

Inflammatory bowel disease (IBD), which includes ulcerative colitis and Crohn’s disease, is a chronic inflammatory disease that is accompanied by abnormalities in the immune system. Although the etiology and pathogenesis of IBD remain largely unknown, multiple risk factors, such as environmental triggers, genetic susceptibility, and alteration of gut microbial flora, have been implicated in its initiation or progression [1, 2]. T cells, which in the presence of various cytokines differentiate into different types of T-helper (Th) cells, including Th1, Th2, Th17, and regulatory T cells (Tregs), are widely accepted to play a major role in the pathogenesis of IBD [3]. An altered Th cytokine network, along with excessive abnormal immune responses, is closely linked to the development of IBD. Biological therapies have been developed with monoclonal antibodies that target Th1 and Th17 cytokines produced by effector T cells, and some of these therapies have proven to be clinically useful for treating patients with IBD and several autoimmune diseases [4].

Prostaglandin (PG) E_2_ is a lipid mediator of many physiological and pathological functions whose production is regulated by the sequential enzymatic pathway involving cyclooxygenase (COX) and PGE synthase (PGES) [5, 6]. On the basis of early studies, COX activity—consisting of constitutive COX-1 and inducible COX-2—had been considered the key step in PGE_2_ synthesis, but subsequent studies discovered that at least three distinct PGES isozymes, cytosolic PGES (cPGES), and microsomal PGES-1 (mPGES-1) and mPGES-2, are responsible for the final step of PGE_2_ synthesis downstream from COX [7–10]. PGE_2_ is known to be a major mediator in gastrointestinal homeostasis and also to be highly produced in the inflamed mucosa of patients with IBD [11]. It is well known to act through 4 kinds of receptor subtypes, EP_1_, EP_2_, EP_3_, and EP_4_, which mediate different signaling [12]. Among the EP receptor subtypes, EP_4_ plays a pivotal role in regulating pathological events in IBD, as well as in maintaining gastrointestinal homeostasis [13–15]. An evaluation of biopsies from patients with IBD showed that both COX-2 expression and PGE_2_ production increase during active phases of the disease [16]. Accordingly, several studies and case reports have implicated nonsteroidal anti-inflammatory drugs (NSAIDs), which inhibit COX activity, in the onset or exacerbation of IBD [17].

One type of PGES, mPGES-1, is an inducible enzyme that acts downstream of COX and specifically catalyzes the conversion of PGH_2_ to PGE_2_ [7, 8]. Several studies in mice lacking mPGES-1 (mPGES-1^−/−^ mice) have provided novel findings on the role of mPGES-1 as a key mediator of many physiological and pathophysiological events in a number of different disease states associated with inflammation and immune response [18–24]. We have previously reported that resistance to bovine type II collagen-induced arthritis in mPGES-1^−/−^ mice is associated with a failure to develop type II collagen-specific antibodies, suggesting an important role of mPGES-1 and its driven PGE_2_ in the development of acquired immune response [25]. We also reported that mPGES-1–driven PGE_2_ facilitates T cell–dependent, antigen-specific humoral responses [26] and also promotes expansion of antigen-specific Th17 and Th1 responses in an autocrine and paracrine fashion [27]. These previous findings strongly suggested the pivotal roles of mPGES-1 in pathogenic T cell immunity.

mPGES-1 protein is overexpressed in inflamed intestinal mucosa of patients with IBD including ulcerative colitis and Crohn’s disease, and mPGES-1 transcription is induced *in vitro* in human colonocytes in response to stimulation with TNFα, a major cytokine implicated in intestinal inflammation in IBD [28], suggesting the importance of mPGES-1 in the pathogenesis of IBD. However, the role of overexpressed mPGES-1 in IBD is still largely unknown.

A dextran sodium sulfate (DSS)–induced colitis model, which is highly dependent on both humoral and cellular immunity, is widely used as a well-established model of IBD [29]. Previous studies have shown that mPGES-1^−/−^ mice are highly susceptible to DSS-induced colitis [30, 31], but the detailed intrinsic mechanisms underlying their susceptibility have not been fully elucidated. The present study demonstrates that mPGES-1 is the main enzyme responsible for colonic PGE_2_ production and exerts anti-colitis activities associated with the suppression of Th17 and Th1 immunologic responses in DSS-induced colitis. Conversely, we also indicate the possible potential for mPGES-1 as a pathogenic factor of colitis by regulating Tregs. Furthermore, our study using T cell depletion suggests the anti-colitis effect of mPGES-1 related to the T cells. Our findings suggest that mPGES-1–driven PGE_2_ has a significant impact on not only the intestinal inflammation but also the pathogenic T cell immunity associated with IBD.

## Materials and methods

### Mice

mPGES-1^−/−^ mice with a C57BL/6 background, originally generated by Prof. Shizuo Akira [22], were purchased from the Oriental Bioservice Inc. (Kyoto, Japan). mPGES-1 heterogeneous mice were mated to generate mPGES-1^−/−^ mice and littermate wild-type (WT) mice. Genotypes were identified by polymerase chain reaction (PCR) analysis of a tail biopsy DNA extract by using specific primers for the mPGES-1^−/−^ allele and WT allele. Mice were housed in cages in a specific pathogen-free barrier facility and were cared for and handled in accordance with the guidelines of the Animal Research and Ethics Committee of Kitasato University and the Safety Committee for Recombinant DNA Experiments of Kitasato University. All animal experiments were approved by the Animal Research and Ethics Committee of Kitasato University (Approval number Ei-ken 19-12), and all experiments in mPGES-1^−/−^ mice were approved by the Safety Committee for Recombinant DNA Experiments of Kitasato University (Approval number 3593).

### Induction of colitis

This study used female mice aged 8 to 12 weeks old. To induce development of colitis, high–molecular-weight, colitis-grade DSS with an average molecular weight of 36,000 to 50,000 (MP Biomedicals, Santa Ana, CA, USA) was added to the drinking water for 7 days at a concentration of either 1 or 2% [32]. Control mice were received plain drinking water without DSS. The severity of colitis was assessed daily by scoring body weight loss, stool consistency, and occult blood in the stool on a scale ranging from 0 (normal) to 4 (severe) and calculating the total disease activity index (DAI) score as the sum of these 3 scores (maximum score: 12), in accordance with a previous report [33]. To evaluate anemia, we measured the number of erythrocytes and concentration of hemoglobin (HGB) and hematocrit (HCT) in peripheral blood by Celltac alpha (Nihon Kohden, Tokyo, Japan).

### Histological assessment of colitis

On day 7 after the start of exposure to DSS, mice were euthanized under anesthesia, and the colons and spleens were collected (we used the adapted Swiss roll technique to collect the colons). Samples were fixed in 4% paraformaldehyde and then embedded in paraffin. Sections of 3.5-μm thickness were stained with hematoxylin and eosin (H&E). Histological analysis of colitis was performed by an observer blinded to the genotypes of the mice. The severity of colitis was rated on the basis of the degree of epithelial damage and inflammatory infiltration. The scores for epithelial damage were as follows: no obvious damage, 0; loss of goblet cells, 1; loss of crypts in the basal one third of the epithelium, 2; loss of crypts in the basal two thirds of the epithelium, 3; and damage to the entire crypt with an intact surface epithelium, 4.

Inflammatory infiltration was scored as follows: no infiltration, 0; infiltration around crypt bases, 1; infiltration reaching the muscularis mucosa, 2; extensive infiltration reaching the muscularis mucosa and thickening of the mucosa with abundant edema, 3; and infiltration of the submucosa, 4. The histological score was calculated as the sum of the epithelial damage and inflammatory infiltration scores (maximum score: 8), in accordance with previous reports [33, 34].

### Epithelial barrier permeability

Intestinal barrier function was assessed with fluorescein isothiocyanate (FITC)-dextran with an average molecular weight of 3000 to 5000 (FD4; Sigma), according to a previous report [35]. Briefly, mice were deprived of food overnight and then FITC-dextran was administered orally (10 mg/mouse at a concentration of 25 mg/mL). After 4 h, blood was immediately collected by cardiac puncture at the time of euthanasia under the anesthesia. The FITC-dextran content in serum was determined by FLUOstar OPTIMA (BGM LABTECH, Offenburg, Germany) with excitation and emission wavelengths of 485 nm and 520 nm, respectively. Dilutions of FITC-dextran were used as a standard curve.

### Real-time PCR analysis

Total RNA was isolated from the colon with a NucleoSpin RNA kit (Macherey-Nagel, Duren, Germany). First-strand cDNAs were synthesized with SuperScript VILO (Thermo Fisher Scientific, Waltham, MA, USA), and then real-time PCR was performed with a Thunderbird SYBR qPCR Mix (Toyobo, Osaka, Japan) in the ABI 7500 Real-Time PCR System (Thermo Fisher Scientific). The primer sets (Eurofins, Luxembourg City, Luxembourg) used in this study are listed in Table 1. The cycling conditions of the PCR reaction were as follows: 1 min at 95 °C, followed by 40 cycles of 15 s each at 95 °C and 1 min at 60°C. The threshold cycle value was normalized by reference to glyceraldehyde 3-phosphate dehydrogenase (GAPDH).

**Table 1:** Primer sequences of various target genes for real-time PCR.

Target gene	Sense primer	Antisense primer
mPGES-1 COX-2 cPGES COX-1 EP_1_ EP_2_ EP_3_ EP_4_ Occludin Claudin-1 IL-17A IFNγ IL-2 TNFα IL-1β IL-6 TGFβ1 IL-23p19 IL-12/23p40 IL-12p35 IL-10 Bak Bid Bim Bad Noxa Bcl2 GAPDH	5’-AGCACACTGCTGGTCATCAA-3’ 5’-AGGACTCTGCTCACGAAGGA-3’ 5’-TGTTTGCGAAAAGGAGAATCCG-3’ 5’-GCCAGAACCAGGGTGTCTGT-3’ 5’-TGCCTCATCCATCACTTC-3’ 5’-TATGCTCCTTGCCTTTCAC-3’ 5’-GCTGTCCGTCTGTTGGTC-3’ 5’-CATCTTACTCATCGCCACC-3’ 5'-AAGCAAGTTAAGGGATCTGC-3' 5'-CCCCATCAATGCCAGGTATG-3' 5’-CAGGGAGAGCTTCATCTGTGT-3’ 5’-CGGCACAGTCATTGAAAGCCTA-3’ 5’-CCTGAGCAGGATGGAGAATTACA-3’ 5’-TCCCCAAAGGGATGAGAAG-3’ 5’-ACTGTGAAATGCCACCTTTTG-3’ 5’-TCCAGTTGCCTTCTTGGGAC-3’ 5’-CTTCAATACGTCAGACATTCGGG-3’ 5’-CCAGCAGCTCTCTCGGAATC-3’ 5’-TGGGAGTACCCTGACTCCTG-3’ 5’-AGTTTGGCCAGGGTCATTCC-3’ 5’-GGTTGCCAAGCCTTATCGGA-3’ 5’-GATGATATTAACCGGCGCTACG-3’ 5’-TAGGCGATGAGATGGACCACAA-3’ 5’-GATCGGAGACGAGTTCAACGAA-3’ 5’-GACGGGCAGCCACCAACAGTCAT-3’ 5’-GTGGAGTGCACCGGACATAACT-3’ 5’-ACAACATCGCCCTGTGGATGAC-3’ 5’-GTCTTCACCACCATGGAGAAGG-3’	5’-CTCCACATCTGGGTCACTCC-3’ 5’-TGACATGGATTGGAACAGCA-3 5’-ACCCATGTGATCCATCATCTCA-3’ 5’-GTAGCCCGTGCGAGTACAATC-3’ 5’-ACCACCAACACCAGCAG-3’ 5’-GACAACAGAGGACTGAGCG-3’ 5’-CCTTCTCCTTTCCCATCTG-3’ 5’-ATGTAAATCCAGGGGTCCA-3’ 5'-CAGATTAGAGTCCAAAGTCA-3' 5'-CACCTCCCAGAAGGCAGAGG-3' 5’-GCTGAGCTTTGAGGGATGAT-3’ 5’-GTTGCTGATGGCCTGATTGTC-3’ 5’-TCCAGAACATGCCGCAGAG-3’ 5’-CACTTGGTGGTTTGCTACGA-3’ 5’-TGTTGATGTGCTGCTGCGAG-3’ 5’-GTGTAATTAAGCCTCCGACTTG-3’ 5’-GTAACGCCAGGAATTGTTGCTA-3’ 5’-CGGATCCTTTGCAAGCAGAA-3’ 5’-GGAACGCACCTTTCTGGTTA-3’ 5’-CAGGTTTCGGGACTGGCTAAGA-3’ 5’-ACCTGCTCCACTGCCTTGCT-3’ 5’-CAGCTGATGCCACTCTTAAATA-3’ 5’-GGAAGGCTGTCTTCACCTAGTC-3’ 5’-TTCTCCATACCAGACGGAAGAT-3’ 5’-AAGGGCTAAGCTCCTCCTCCAT-3’ 5’-TGAGCACACTCGTCCTTCAAGT-3’ 5’-CAGAGACAGCCAGGAGAAATCA-3’ 5’-TCATGGATGACCTTGGCCAG-3’

### Western blot analysis

The tissues were homogenized and lysed in a buffer containing 40 mmol/L Tris/HCl (pH 7.4), 150 mmol/L NaCl, 2 mmol/L EDTA, 1 mmol/L dithiothreitol, 1% Triton X-100, 2 mmol/L sodium orthovanadate, 10 mmol/L NaF, and 10 mmol/L sodium pyrophosphate supplemented with a protease inhibitor cocktail mixture (Sigma, St Louis, MO, USA). Protein contents were measured by a BCA protein assay kit (Thermo Fisher Scientific, Waltham, MA, USA), and bovine serum albumin was used as a standard. Samples were separated by sodium dodecyl sulfate-polyacrylamide gel electrophoresis, and then proteins were transferred onto an Amersham Hybond PVDF membrane (GE Healthcare, Little Chalfont, UK). After a blocking procedure, the membrane was incubated with anti-mPGES-1 (No. 160140; Cayman Chemicals, Ann Arbor, MI, USA), anti-cPGES (No. 160150; Cayman Chemicals), anti-COX-2 (No. 160106; Cayman Chemicals), anti-COX-1 (NAB37401; R&D Systems, Minneapolis, MN, USA), or anti-β-actin (clone 2F3; Fujifilm Wako Pure Chemical, Osaka, Japan) antibody and then incubated with a secondary antibody coupled to horseradish peroxidase (Jackson ImmunoResearch Laboratories, PA, USA). After washing, protein was detected by enhanced chemiluminescence (GE Healthcare, Little Chalfont, UK).

### Measurement of PGE_2_ and PGD_2_

Tissues were homogenized in 70% methanol supplemented with 30 μM indometacin. The homogenates were centrifuged at 15,000 *g* at 4 °C for 20 min. The supernatant was evaporated under a nitrogen gas stream and suspended in enzyme immunoassay buffer, and the levels of PGE_2_ and PGD_2_ as a MOX-PGD_2_ (a stable metabolite of PGD_2_) were measured by enzyme-linked immunosorbent assay kits (Cayman Chemicals, Ann Arbor, MI, USA), according to the manufacturer’s protocol [36]. Optical density was measured with the Benchmark microplate reader (Biorad, Hercules, CA, USA).

### Immunofluorescence double staining

Colons (obtained by the adapted Swiss roll technique) were embedded in OCT compound, snap-frozen, and stored at – 80 °C. Cryostat sections (10 μm) were fixed in cold acetone and stained with an anti-mPGES-1 monoclonal antibody (ab180589, Abcam, Cambridge, UK) and Alexa Fluor 594-conjugated anti-E-cadherin (clone DECMA-1, a marker of epithelial cells; BioLegend, San Diego, CA, USA), Alexa Fluor 594-conjugated anti-CD3 (clone 17A2, a marker of T cells; BioLegend) or Alexa Fluor 594-conjugated anti-CD11b (clone M1/70, a marker of monocytes/macrophages; BioLegend). For the staining of mPGES-1, sections were followed by incubation with Alexa Fluor 488-conjugated secondary antibody (Jackson ImmunoResearch) (West Grove, PA, USA). Color images were obtained by BX51 fluorescence microscope (Olympus Corporation, Tokyo, Japan).

### Isolation of lamina propria mononuclear cells and splenocytes

Lamina propria mononuclear cells (LPMCs) were isolated according to the modified method in a previous report [37]. Briefly, colon tissues were obtained from WT and mPGES-1^−/−^ mice on day 7 after the start of exposure to DSS, washed with cold PBS and cut into 1-cm pieces. The pieces were treated with 5 mM EDTA and 1 mM dithiothreitol in Hanks balanced salt solution to remove the epithelial cells, and then the residues were digested with 1.5 mg/mL collagenase D (Roche diagnostics, Rotkreuz, Switzerland) and 0.05 mg/mL DNase I (Roche diagnostics). The dispersed cells were separated in a Percoll gradient to obtain LPMCs. Splenocytes were also isolated, as described in a previous study [26].

### Flow cytometry (FCM) analysis

LPMCs and splenocytes were incubated with anti-CD16/32 antibody (TruStain fcX; BioLegend) to block FcγII/III receptor–mediated nonspecific antibody binding before surface staining of cell surface markers. Cells were then stained with fluorochrome-conjugated anti-mouse monoclonal antibodies (BioLegend) against CD3 (clone 17A2) and CD4 (clone GK1.5) before intracellular staining for interferon-γ (IFNγ) and IL-17A. Isotype controls were also used to characterize the background signal from off-target antibody binding. The Zombie Aqua Fixable Viability kit (BioLegend) was used in all analyses to remove dead cells and avoid background or unspecific staining of dead cells. For staining of IL-17A- and IFNγ-producing T cells, intracellular staining for IFNγ (clones XMG1.2; Biolegend) and IL-17A (TC11-18H10.1; Biolegend) was performed after stimulation of cells, staining of surface molecules, and fixation and permeabilization of cells. Briefly, single-cell suspensions were incubated with phorbol 12-myristate 13-acetate (50 ng/mL, Sigma), ionomycin (500 ng/mL, Sigma), and GolgiStop (BD PharMingen, Franklin Lakes, NJ) for 4 h *in vitro* in RPMI1640 supplemented with 10% fetal bovine serum, penicillin/streptomycin, and freshly added 50 μmol/L 2-mercaptoethanol, as described in a previous report [27]. The Cytofix/Cytoperm Plus Fixation/Permeabilization kit (BD PharMingen) was used to fix, permeabilize, and stain cells, in accordance with the manufacturer’s instructions. Tregs were detected with the Treg Detection kit (Miltenyi Biotec, Bergisch Gladbach, Germany) with CD3 antibody (clone 17A2), in accordance with the manufacturer’s instructions. The stained cells were analyzed by a MACS Quant Analyzer (Miltenyi Biotec). The gating strategy was always in accordance with the following hierarchy: total events → lymphocyte gate (FSC-A/SSC-A) → living cells (Live/Dead−) → CD3^+^CD4^+^, with subsequent gating indicated in each experiment.

### *In vivo* CD4 positive T cell depletion

For CD4^+^ T cell depletion experiments, mice are treated with 0.1 mg of anti-CD4 monoclonal antibody (clone GK1.5; Bio X Cell, West Lebanon, New Hampshire) or 0.1 mg of isotype-matched control antibody (clone LTF-2; Bio X Cell) *via* intraperitoneal injections on the days − 1 and + 3 relative to the start of DSS administration [38]. Depletion of CD4^+^ T cell was confirmed by FCM analysis of T cell population in the peripheral blood and spleen by staining with fluorochrome-conjugated anti-mouse monoclonal antibodies (BioLegend) against CD3 (clone 17A2) and CD4 (clone RM4.4), CD8 (clone 53-6.7) and CD11b (clone M1/70).

### *In situ* apoptosis detection

Apoptotic cells were detected by *in situ* apoptosis detection kit (Takara Bio Inc, Shiga, Japan), according to the manufacturer’s protocol. Briefly, paraffin-embedded colon sections of 3.5-μm thickness (obtained by the adapted Swiss roll technique) were incubated with a terminal dexoynucleotidyl transferase enzyme and then with an anti-FITC peroxidase-conjugated secondary antibody. The positive signals were visualized with 3,3′-diaminobenzidine (Takara DAB substrate: Takara Bio Inc, Shiga, Japan), and then sections were counterstained with methyl green.

### Statistical analysis

Data were expressed as the means + SEM. Statistical analysis was performed with the Sigmastat 3.5 software (Systat Software, Inc., San Jose, CA, USA). Data from more than two groups were compared by 2-way analysis of variance (ANOVA) followed by Tukey multiple comparison test, and data from 2 groups were compared by *t* test after testing for normal distribution. *P* < 0.05 was considered statistically significant.

## Results

### Exacerbated DSS-induced colitis in mice with mPGES-1 genetic deletion

**Fig. 1:**
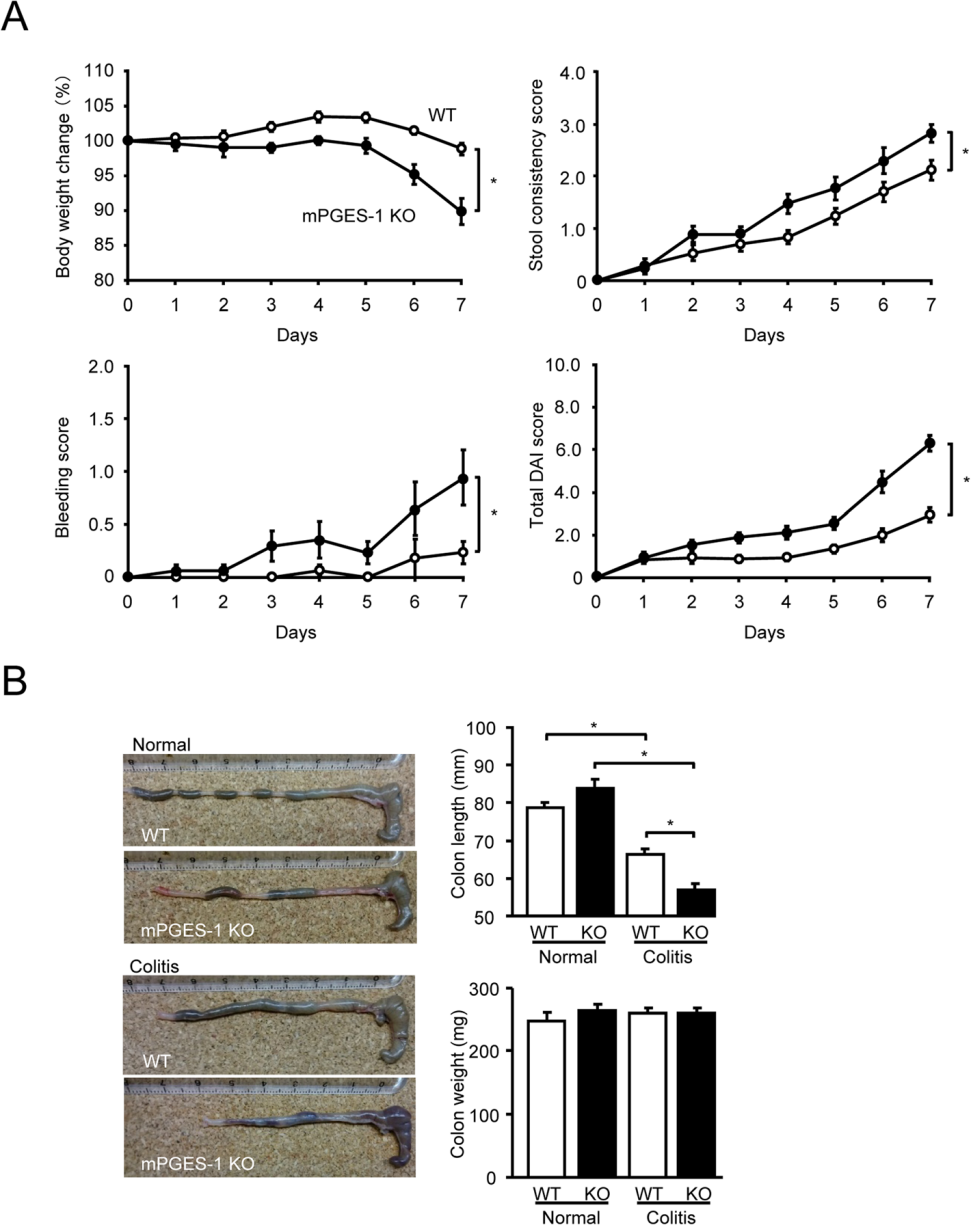
Clinical course of DSS-induced colitis in mice with a mPGES-1 genetic deletion. **A** Time course of change in body weight, stool consistency, bleeding score, and total disease activity index (DAI) score of WT and mPGES-1^−/−^ mice after indicated days of exposure to 1% DSS (*n* = 17). **B** On day 7 after the start of exposure to 1% DSS, the length and weight of the colon were measured as an indirect marker of inflammation (*n* = 3 to 17). Pictures of the colon are representative examples in WT and mPGES-1^−/−^ mice. *P < 0.05; 2-way ANOVA followed by Tukey multiple comparison test

mPGES-1^−/−^ and WT mice were given a relatively low dose of 1% DSS for 7 days, and the severity of colitis was daily evaluated for a week. As shown in Fig. 1A, after 7 days’ administration of 1% DSS, body weight was significantly lower in the mPGES-1^−/−^ mice than in the WT mice. In addition, both genotypes of 1% DSS–treated mice exhibited diarrhea and fecal bleeding, but these colitis symptoms were more severe in mPGES-1^−/−^ mice than in WT mice. The total DAI colitis scores were also markedly higher in mPGES-1^−/−^ mice than in WT mice, as were the separate scores for stool consistency and bleeding. Weekly food uptake was significantly lower in mPGES-1^−/−^ mice than in WT mice. Uptake of DSS-containing water did not differ between WT and mPGES-1^−/−^ mice, although the latter showed a trend towards consuming less water (Fig. S1). Experiments were mainly performed by using female mice in this study, but the total DIA scores of male mPGES-1^−/−^ mice were also significantly higher in male WT mice after 7 days’ administration of 1% DSS (WT: 5.5 + 0.6 (*n* = 4), mPGES-1^−/−^: 8.4 + 0.5 (*n* = 5) on Day 7 (*P < 0.05; *t* test)).

Colon shortening has been proven to be a useful inflammatory marker and indicator of colitis [39]. Consistent with the total DAI score, by day 7, both genotypes of DSS-treated mice showed colon shortening, but the colon was significantly shorter in the mPGES-1^−/−^ mice than in the WT mice (Fig. 1B). No significant differences in colon weight were observed between the genotypes and DSS administered.

Notably, only approximately 60% of mPGES-1^−/−^ mice (8 of 13 mice) survived after 7 days’ exposure to the higher dose of 2% DSS, but all WT mice (12 of 12 mice) survived. Thus, the subsequent experiments in the 2% DSS-treated mPGES-1^−/−^ mice were continued in only survived mice until day 7 of exposure to DSS.

### Histological features of DSS-induced colitis in mPGES-1^−/−^ mice

**Fig. 2:**
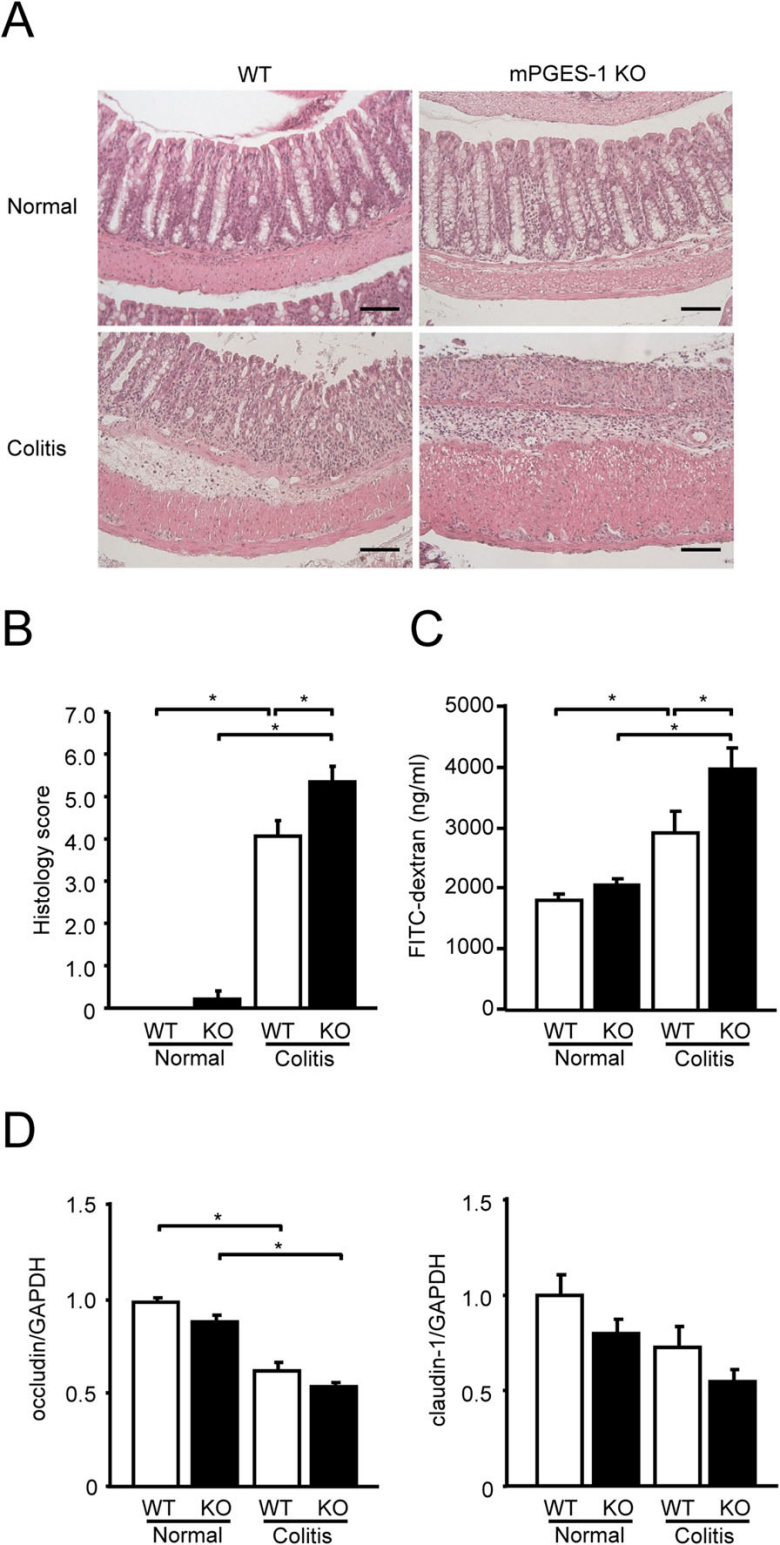
Histological analysis of DSS-induced colitis in mPGES-1^−/−^ mice. **A** Colons of mPGES-1 WT and mPGES-1^−/−^ mice were collected on day 7 after the start of exposure to 1% DSS, and sections were stained with H&E. Results are representative examples adapted with the Swiss roll technique in WT and mPGES-1^−/−^ mice (*n* = 5 to 17). Scale bar, 100 μm. **B** Histological scores in WT and mPGES-1^−/−^ mice (*n* = 5 to 17). Colon sections were examined by a blinded researcher, who calculated the epithelial damage score and inflammatory infiltration score and summed the 2 scores (maximum score: 8). **C** Intestinal permeability was assessed with FITC-dextran on day 7 after the start of exposure to 1% DSS (*n* = 9 to 17). *P < 0.05; 2-way ANOVA followed by Tukey multiple comparison test. **D** Expression of mRNA for the tight junction molecules occludin and claudin-1 in colon from mice treated or not treated with 1% DSS for 7 days were analyzed by real-time RT-PCR. Levels of mRNA expression are shown as the fold induction relative to the expression in WT mice without DSS administration (assigned the value “1”). *P < 0.05; 2-way ANOVA followed by Tukey multiple comparison test (*n* = 7 to 10)

Severity of colitis was further assessed by histological evaluation with H&E-stained colon sections. As shown in Fig. 2A, after DSS administration, the colons of WT mice showed the characteristic features of colitis, with extensive areas of mononuclear infiltrates, focal crypt epithelial destruction, and edema. Compared with WT mice, mPGES-1^−/−^ mice showed greater epithelial damage and infiltration of inflammatory cells. After grading of these histological features by an observer blinded to the genotypes of the sections, the sum of the epithelial damage and inflammatory infiltration scores was significantly higher in mPGES-1^−/−^ mice than in WT mice (Fig. 2B).

To characterize the protective effect of mPGES-1 on the epithelial layer in DSS-treated mice, we quantified the permeability by orally administering FITC-dextran to mice and measuring the serum levels. On day 7 after the start of DSS administration, significantly more FITC-dextran diffused through the epithelium in mPGES-1^−/−^ mice than in WT mice (Fig. 2C). DSS treatment resulted in a decrease in the colonic mRNA expression level of the tight junction molecules occludin and claudin-1, which play crucial roles in regulating intestinal paracellular permeability [40], but the differences in the expression levels of these molecules between WT and mPGES-1^−/−^ mice did not reach statistical significance in this study (Fig. 2D).

### mPGES-1^−/−^ mice display anemia and extramedullary hematopoiesis in spleen

**Fig. 3:**
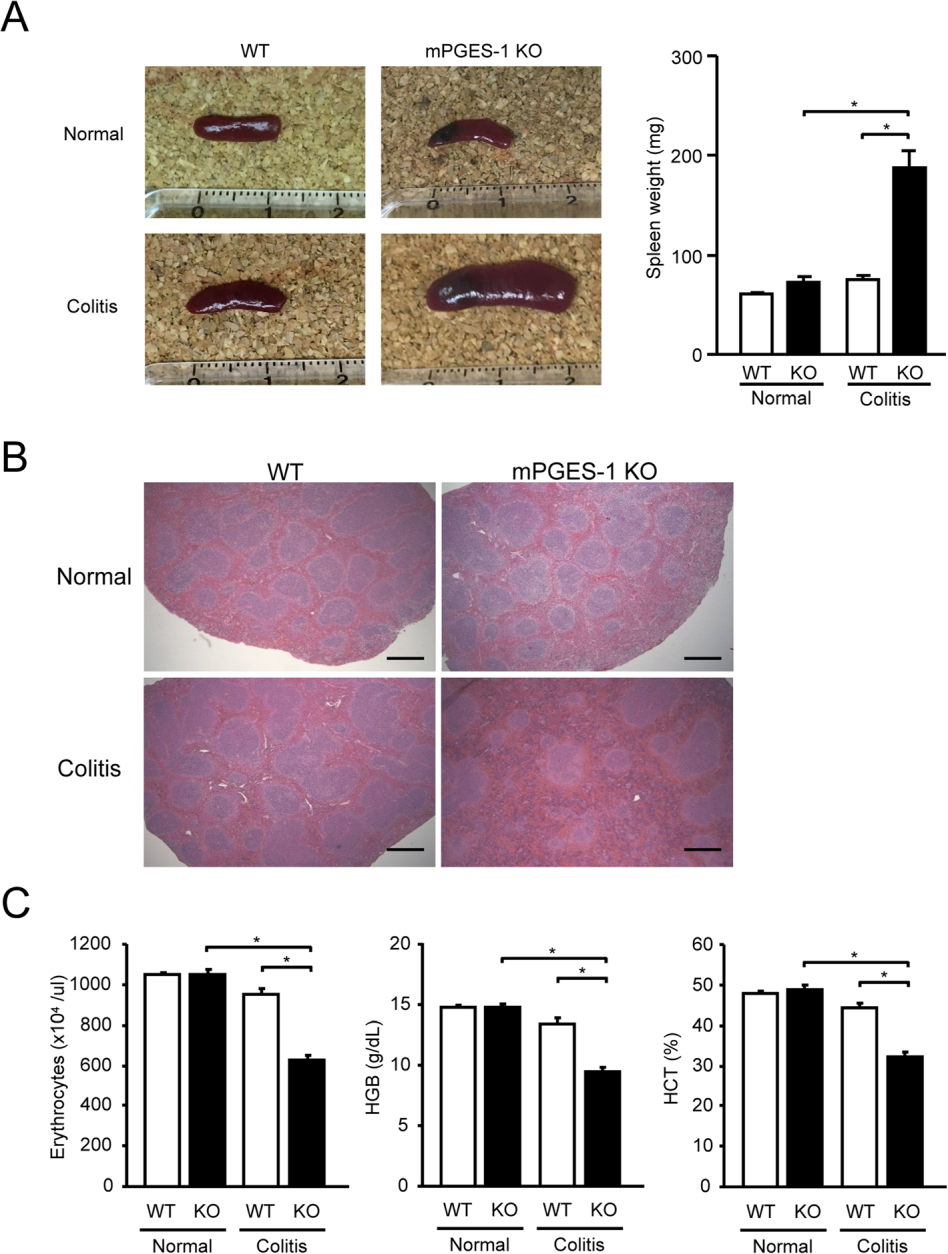
Analysis of splenomegaly and anemia in mPGES-1^−/−^ mice. **A** Spleen was isolated, imaged and weighed on day 7 after the start of exposure to 1% DSS. **B** Also on day 7, morphological analysis of the spleen was performed by H&E staining. Representative images are shown. Scale bar, 500 μm. **C** Erythrocyte count, hemoglobin (HGB) concentration, and hematocrit (HCT) in the peripheral blood were measured on day 7 after the start exposure to 1% DSS. *P < 0.05; 2-way ANOVA followed by Tukey multiple comparison test (*n* = 3 to 17)

mPGES-1^−/−^ mice exhibited marked splenomegaly by day 7 after the start of DSS administration, as shown in Fig. 3A. The weight of the spleens indicated that the spleens of mPGES-1^−/−^ mice were approximately 2.5-fold larger after administration of DSS, but the spleens of WT mice were not affected by DSS. In mPGES-1^−/−^ mice, histological examination of the spleen after DSS administration showed massive expansion of the red pulp along with increased cellularity compared with WT mice (Fig. 3B). The expansion of splenic red pulp, along with increased cellularity, represents a key feature of enhanced extramedullary hematopoiesis in the spleen [41, 42] and also generally represents a valid cause of an enlarged spleen with extramedullary hematopoiesis [43, 44]. In severe colitis after long-term, repeated DSS treatment, splenic extramedullary hematopoiesis is increased, as reflected by increases in spleen weights and the red pulp, resulting in increased reticulocyte counts in response to anemia [45]. Indeed, mPGES-1^−/−^ mice showed significantly decreased erythrocytes and lower levels of HGB and HCT in peripheral blood, indicating severe anemia after DSS administration (Fig. 3C). The splenomegaly and extramedullary hematopoiesis might be linked with the anemia associated with gastrointestinal hemorrhage during severe colitis in the absence of mPGES-1.

In the subpopulation analysis of splenocytes from mice with colitis, we found no differences in the number of CD19^+^, CD11c^+^, CD11b^+^, or Gr-1^+^ cells between WT and mPGES-1^−/−^ mice (Table S1). We did not detect any evidence of enhanced immunity in the enlarged spleen of mPGES-1^−/−^ mice with colitis.

### Induction of mRNA expression for mPGES-1 and COX-2 in DSS-induced colitis

**Fig. 4:**
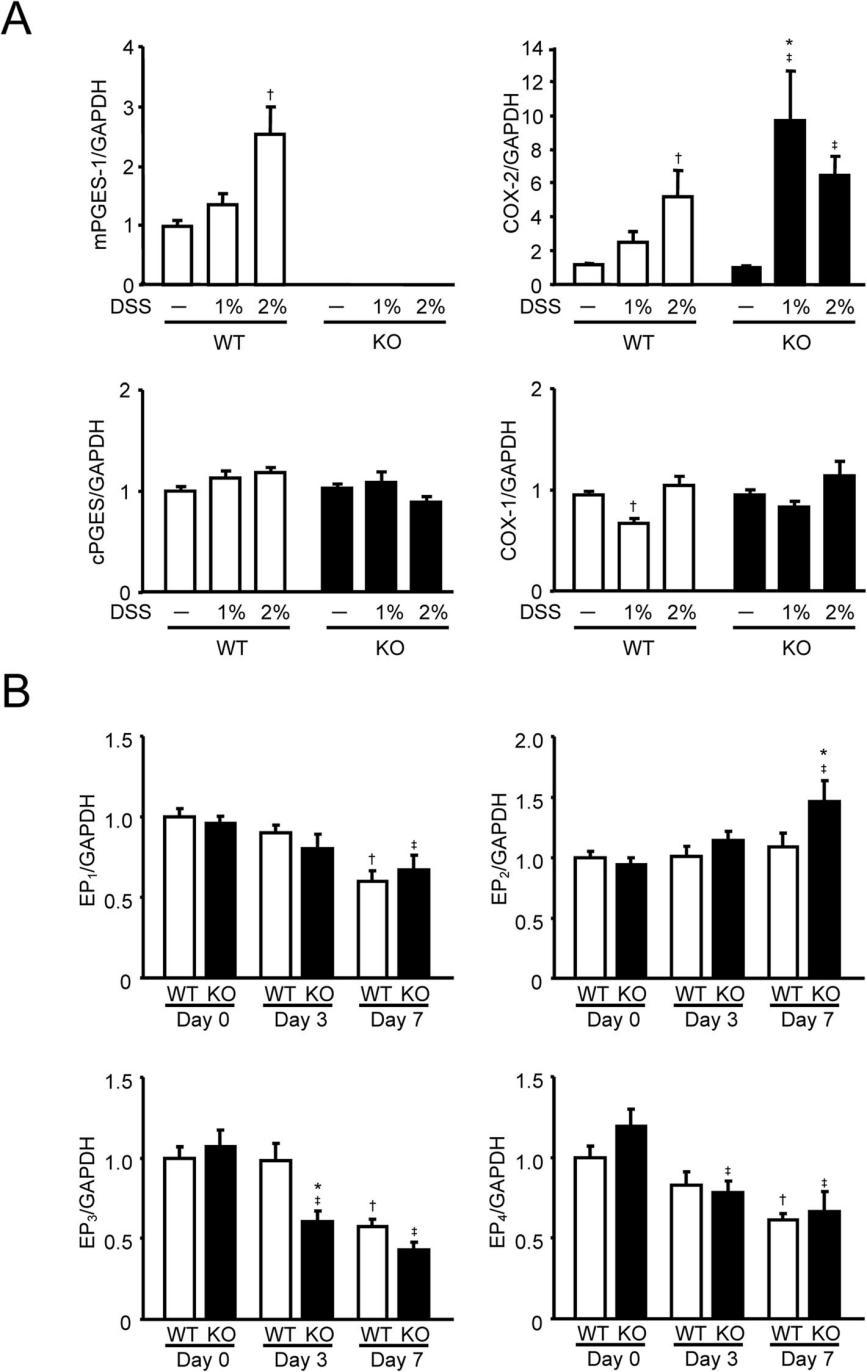
Expressions of mRNA for PGE_2_ biosynthetic enzymes and EP receptor subtypes in colon after exposure to DSS. **A** Expression of mRNA for PGES and COX isozymes in colon from mice treated or not treated with the indicated dose of DSS for 7 days were analyzed by real-time RT-PCR (*n* = 7 to 12). Levels of mRNA expression are shown as the fold induction relative to the expression in WT mice without DSS administration (assigned the value “1”). *P < 0.05 vs WT mice within each day, ^†^*P* < 0.05 vs non-DSS-treated WT mice, and ^‡^*P* < 0.05 vs non-DSS-treated KO mice; 2-way ANOVA followed by Tukey multiple comparison test. **B** Expression of EP receptor mRNA in colon from mice treated with 1% DSS for indicated days was analyzed by real-time RT-PCR (*n* = 7 to 10). Levels of mRNA expression are shown as the fold induction relative to day 0 expression in WT (assigned the value “1”). *P < 0.05 vs WT mice within each day, ^†^*P* < 0.05 vs WT at day 0, and ^‡^*P* < 0.05 vs KO mice at day 0; 2-way ANOVA followed by Tukey multiple comparison test

Because we observed greater severity of DSS-induced colitis in mPGES-1^−/−^ mice, we next determined the mRNA expression of PG biosynthetic enzymes in the colon from WT and mPGES-1^−/−^ mice with or without DSS administration (Fig. 4A). In WT mice, colonic expression of mPGES-1 mRNA was basally detectable and significantly increased in a dose-dependent manner at 7 days after the start of DSS administration. As expected, mPGES-1 expression was completely abolished in mPGES-1^−/−^ mice either with or without DSS administration. The colonic expression of cPGES, a constitutive isozyme of PGES, was not changed by DSS treatment in either the WT or mPGES-1^−**/−**^ mice. Colonic expression of COX-2 mRNA was significantly increased by DSS in both WT and mPGES-1^−/−^ mice in a concentration-dependent manner, and the level of COX-2 expression in mPGES-1^−/−^ mice was significantly higher than in WT mice. COX-1 was also expressed in the colon, but the expression level was similar in both mPGES-1^−/−^ and WT mice.

After DSS administration, the expressions of EP_1_, EP_3_, and EP_4_ decreased significantly over time in both WT and mPGES-1^−/−^ mice, but the expression of EP_3_ decreased faster in mPGES-1^−/−^ mice than in WT mice (Fig. 4B). In addition, the mPGES-1^−/−^ mice showed increased EP_2_ expression on day 7 after the start of DSS administration.

### Expression and localization of mPGES-1 and prostanoid production in the colon

**Fig. 5:**
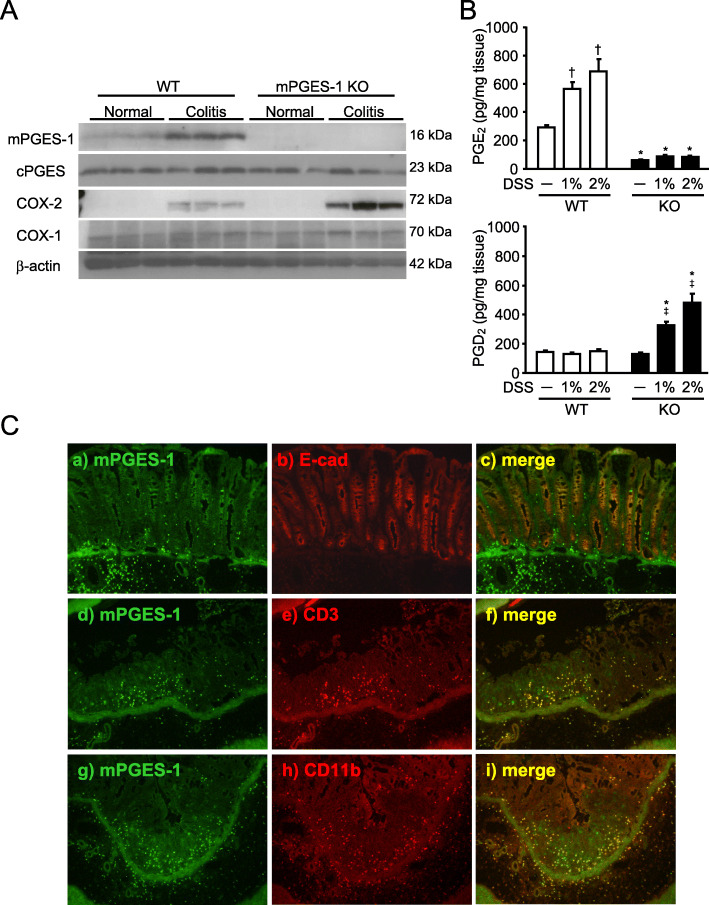
mPGES-1 protein expression and prostanoid production in the colon with colitis by DSS. **A** Expression of protein for PGES and COX isozymes in colon on day 7 after the start of exposure to 1% DSS were analyzed by western blot analysis (*n* = 3). **B** The levels of PGE_2_ and PGD_2_ in the colon from mice treated or not treated with the indicated dose of DSS for 7 days were measured by ELISA. *P < 0.05 vs WT mice within each day, ^†^*P* < 0.05 vs non-DSS-treated WT mice, and ^‡^*P* < 0.05 vs non-DSS-treated KO mice; 2-way ANOVA followed by Turkey multiple comparison test (*n* = 3 to 5). **C** Representative double immunofluorescence staining image of Swiss-roll colon sections of WT mice on day 7 after the start of exposure to 1% DSS. Double staining for mPGES-1 (green) and E-cadherin, CD3 or CD11b (red) showed mPGES-1 immunoreactivity mostly colocalized with an epithelial cell marker, E-cadherin, a T cell marker CD3 and a monocytes/macrophage marker CD11b in the colon

We next examined the protein expression of PGE_2_ biosynthetic enzymes in the colon (Fig. 5A). The colon of WT mice basally expressed a low level of mPGES-1 protein, and expression of mPGES-1 was upregulated in response to DSS (Fig. 5A). As expected, the colon of mPGES-1^−/−^ mice did not express mPGES-1 protein, either with or without DSS administration. In addition, COX-2 protein was not detected without DSS administration, but it was significantly induced by DSS in both WT and mPGES-1^−/−^ mice. The induction of COX-2 was greater in mPGES-1^−/−^ mice than in WT mice. On the other hand, cPGES and COX-1 protein were present without DSS administration in both WT and mPGES-1^−/−^ mice, and the expression of these proteins did not change after induction of colitis. These results were well consistent with the mRNA expression patterns of each enzyme.

We next determined the role of mPGES-1 in colonic prostanoid production under normal and colitis state. As shown in Fig. 5B, the basal level of colonic PGE_2_ without induction of colitis was low in WT mice. After DSS administration, the level of colonic PGE_2_ increased significantly in WT mice in a concentration-dependent manner but did not increase in mPGES-1^−/−^ mice. Notably, even in non-inflamed colon without DSS, mPGES-1 genetic deletion resulted in greater reduction of colonic PGE_2_ when compared with WT mice. These data clearly indicate that mPGES-1 is the main synthase responsible for colonic PGE_2_ production not only in colitis but also in the healthy condition. A minimal level of PGE_2_ was still detectable in mice without mPGES-1, indicating that cPGES and other PGES isozymes other than mPGES-1 contribute somewhat to colonic PGE_2_ production.

To further determine whether there was compensatory shunting of arachidonic acid into other prostanoid pathways in the mPGES-1^−/−^ mice, we also measured the level of colonic PGD_2_, known as an anti-colitis prostanoid [16, 46]. WT and mPGES-1^−/−^ mice displayed similar baseline levels of PGD_2_ in the colon; however, DSS administration resulted in a marked increase of colonic PGD_2_ production in mPGES-1^−/−^ mice but not in WT mice (Fig. 5B).

In our immunohistochemical analysis of WT mice to identify the site responsible for PGE_2_ production in the colon during colitis, we detected mPGES-1 fluorescence immunoreactivity in the colonic mucosal epithelium and infiltrated inflammatory cells in underlying connective tissues and the lamina propria (LP) of WT mice (Fig. 5C). Double staining for mPGES-1 (green) and E-cadherin, CD3 or CD11b (red), showed mPGES-1 immunoreactivity mostly colocalized with immunoreactivity of an epithelial cell marker, E-cadherin, a T cell marker CD3 and a monocytes/macrophage marker CD11b in the colon. In the negative control, which used the frozen colon section of mPGES-1^−/−^ mice, we did not detect immunoreactivity in the colonic mucosal epithelium and infiltrated inflammatory cells in underlying connective tissues and the LP, but likely nonspecific immunoreactivity was detected only in the colonic muscular layer (data not shown). These data suggest that the overexpression of mPGES-1 in the mucosal epithelium, immune cells, and inflammatory cells at inflammatory sites could be responsible for elevated PGE_2_ production in the colon during colitis.

### Facilitation of colonic Th17/Th1-related cytokine expression in mPGES-1 deficiency

**Fig. 6:**
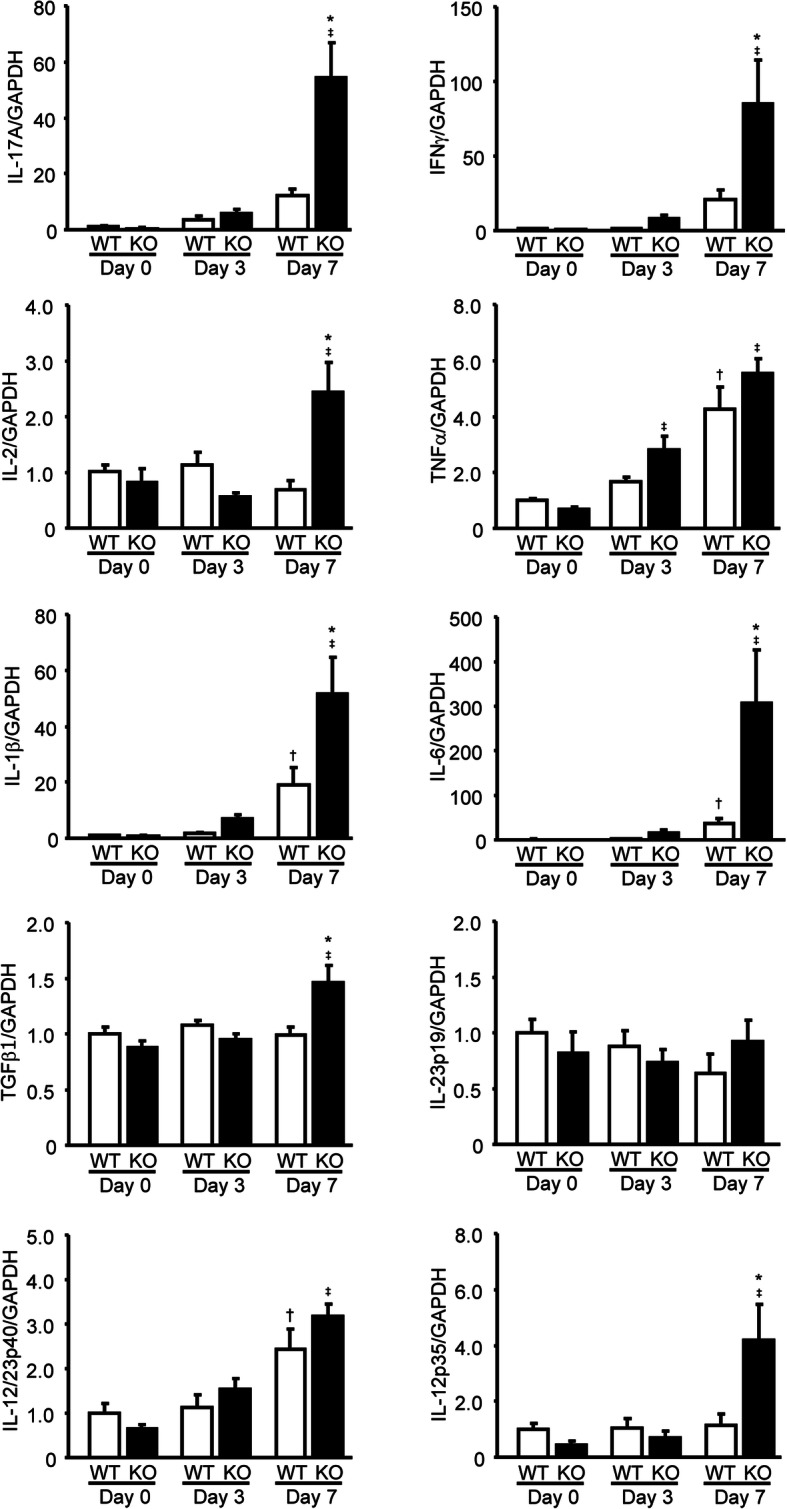
Colonic expression profile of Th17/Th1-related cytokines in mPGES-1^−/−^ mice with DSS-induced colitis. Expressions of mRNA for IL-17A, IFNγ, IL-2, TNFα, IL-1β, IL-6, TGFβ1, IL-23p19, IL-12/23p40, and IL-12p35 in colon from mice treated with 1% DSS for indicated days were analyzed by real-time RT-PCR (*n* = 7 to 10). Levels of mRNA expression are shown as the fold induction relative to day 0 expression in WT mice (assigned the value “1”). *P < 0.05 vs WT mice within each day, ^†^*P* < 0.05 vs non-DSS-treated WT mice, and ^‡^*P* < 0.05 vs non-DSS-treated KO mice; 2-way ANOVA followed by Tukey multiple comparison test

To clarify the mechanism how mPGES-1 exerts protective effect on the colitis, we next turned our attention to a T cell immunologic response which is an essential event in experimental colitis as well as IBD. As shown in Fig. 6, the colons of mPGES-1^−**/−**^ mice expressed markedly higher levels of mRNA for IL-17A (Th17 cytokine) and IFNγ and IL-2 (Th1 cytokines) than the colons of WT mice after 7 days’ administration of 1% DSS. Notably, after treatment with high dose of 2% DSS the colonic expressions of IL-17A and IFNγ mRNA were also higher in mPGES-1^−/−^ mice compared with those in WT mice (IL-17: WT 112.6 + 23.9 (*n* = 12), KO 210.1 + 60.7 (*n* = 8); IFNγ: WT 16.4 + 6.7 (*n* = 12), KO 46.5 + 19.5 (*n* = 8)). In addition, the colonic expressions of the major proinflammatory cytokines IL-1β and IL-6, which are also known to be essential for Th17 cell differentiation from naive CD4^+^ T cells [47, 48], were higher in mPGES-1^−**/−**^ mice. The expression level of TGFβ1, an essential inducer of Th17 cell differentiation in combination with IL-6 [49], was also higher in mPGES-1^−**/−**^ mice than in WT mice. In contrast, the levels of components of IL-23 (IL-23p19 and IL-12/23p40), which were shown to be a requirement for human Th17 differentiation [50], did not differ between WT and mPGES-1^−**/−**^ mice. The expression of IL-12p35, a subunit of IL-12 that was reported as being required for IFNγ-producing Th1 differentiation [51], was significantly higher in mPGES-1^−**/−**^ mice than in WT mice. These results correlated well with the pathophysiological and histopathological evidence of colitis observed under mPGES-1 deficiency in the present study. Taken together, the results suggest that mPGES-1–driven PGE_2_ suppresses the excessive abnormal immune responses associated with the Th17/Th1-related cytokine during colitis. As unexpected, the colonic expression levels of TNFα, one of the major proinflammatory cytokines relevant to IBD, did not differ between WT and mPGES-1^−**/−**^ mice.

### Genetic deletion of mPGES-1 results in enhancing generation of IL-17A– and IFNγ–producing T cells

**Fig. 7:**
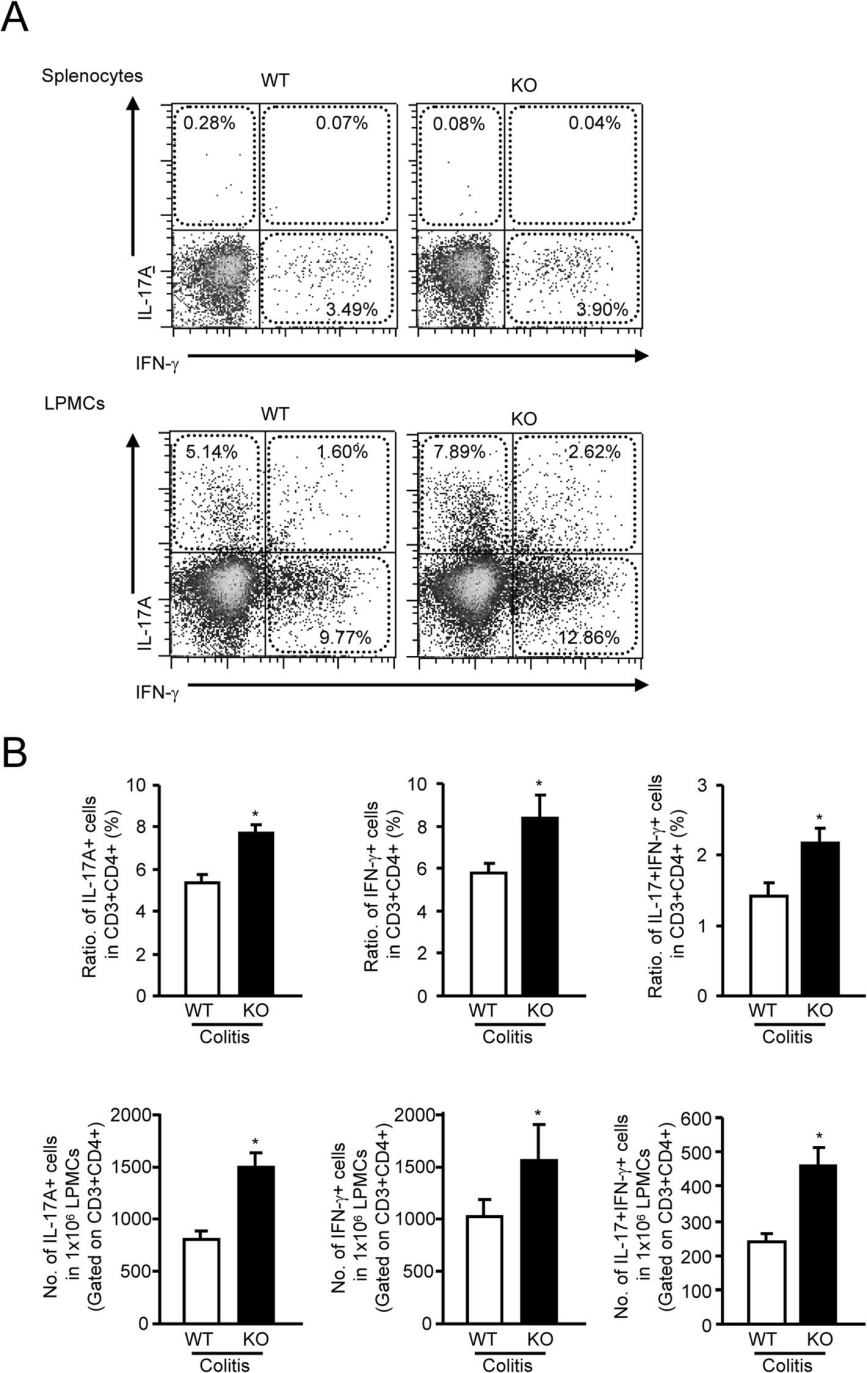
Generation of IL-17A- and IFNγ-producing T cells in colonic LPMCs and splenocytes of mPGES-1^−/−^ mice with DSS-induced colitis. **A** Representative FCM plot of IL-17A-producing Th17 cells and IFNγ-producing Th1 cells in CD3^+^CD4^+^ T cells of splenocytes and colonic LPMCs isolated from WT and mPGES-1^−/−^ mice with colitis. Colonic LPMCs were pooled from 4 mice in each experiment on day 7 after the start of exposure to 1% DSS and analyzed by FCM, as described in the Methods (*n* = 5). **B** The ratio and the number of IL-17A^+^ and IFNγ^+^ cells in CD3^+^CD4^+^ T cells of colonic LPMCs on day 7 after the start of exposure to 1% DSS (*n* = 5). *P < 0.05 vs WT; *t* test

To further determine the impact of mPGES-1 on the developing Th17 and Th1 immunologic responses associated with colitis, after inducing colitis, we determined the fraction of Th17 and Th1 cells that produced IL-17A and IFNγ in cell populations from spleen and LP of the colon from mPGES-1^−**/−**^ and WT mice. Cells were isolated during colitis, stimulated *ex vivo* with phorbol 12-myristate 13-acetate/ionomycin, then surface stained for CD3/CD4, fixed, stained for intracellular IL-17A and IFNγ and analyzed by FCM. As shown in Fig. 7A, in splenocytes in both mPGES-1^−**/−**^ and WT mice, we found few Th17 cells that can produce IL-17A. The percentage of IFNγ-producing Th1 cells in splenocytes was also similar in both genotypes. In addition, the IL-17^+^IFNγ^+^ double positive cells in splenocytes were few in both mPGES-1^−**/−**^ and WT mice. However, in the LPMCs from the colon the populations of IL-17A-producing Th17 cells, IFNγ-producing Th1 cells and L-17 and IFNγ double-producing cells within CD3^+^CD4^+^ cells were larger in mPGES-1^−**/−**^ mice than in WT mice (Fig. 7A and B). Compared with WT mice, the numbers of both CD3^+^CD4^+^ Th17 cells and CD3^+^CD4^+^ Th1 cells within LPMCs were also significantly increased in the colonic LP from mPGES-1^−**/−**^ mice (Fig. 7B), which was consistent with facilitation of colonic Th17/Th1-related cytokine expression under mPGES-1 deficiency (Fig. 6).

### Increased Treg population and IL-10 expression in mPGES-1^−/−^ mice during DSS-induced colitis

**Fig. 8:**
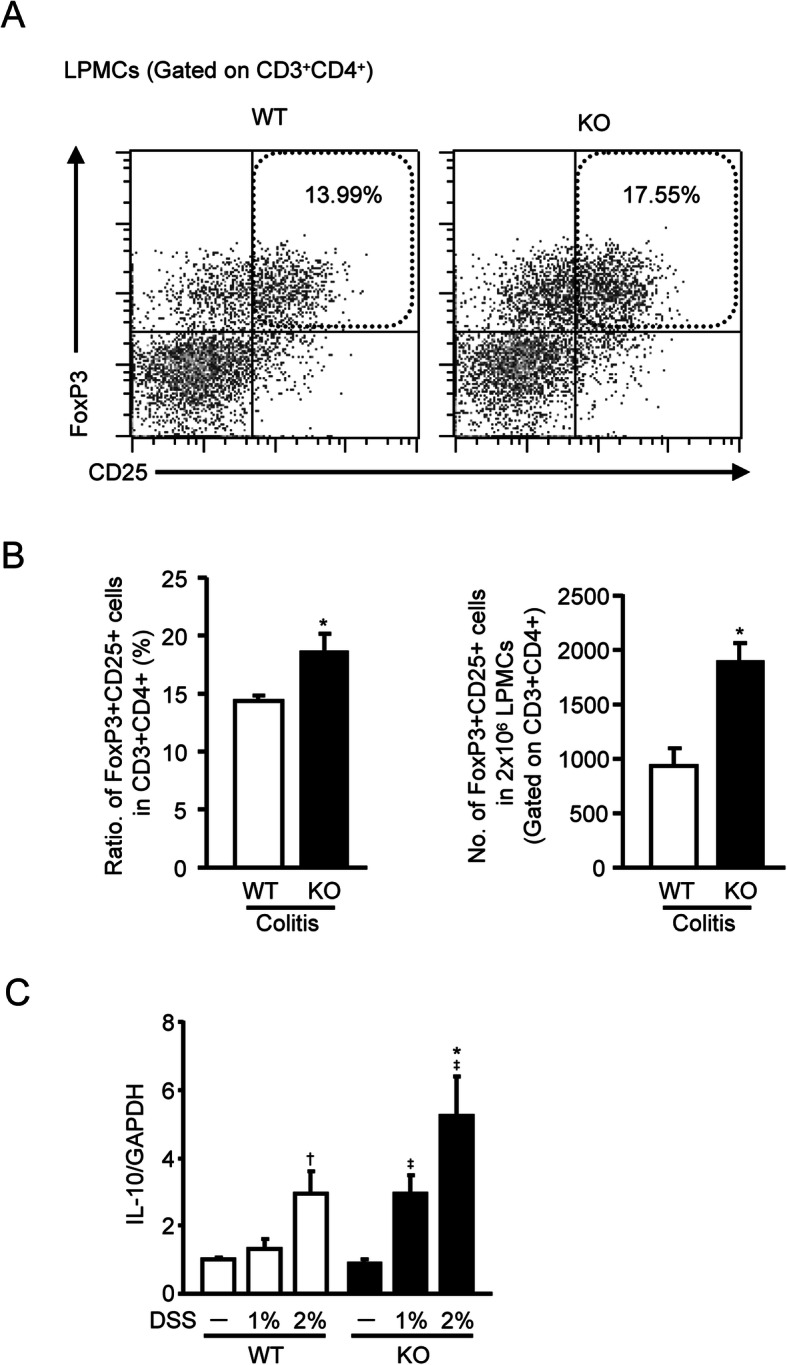
Effect of mPGES-1 gene deletion on the generation of Tregs in DSS-induced colitis. **A** Representative FCM plot of FoxP3^+^CD25^+^ Tregs in CD3^+^CD4^+^ T cells of colonic LPMCs, isolated from WT and mPGES-1^−/−^ mice with colitis. Colonic LPMCs were pooled from 4 mice in each experiment on day 7 after the start of exposure to 1% DSS and subjected to FCM analysis, as described in the Methods (*n* = 5). **B** The ratio and the number of FoxP3^+^CD25^+^ cells in CD3^+^CD4^+^ T cells of colonic LPMCs on day 7 after the start of exposure to 1% DSS (*n* = 5). **C** Expression of mRNA for IL-10 in the colon from mice treated or not treated with the indicated dose of DSS for 7 days was analyzed by real-time RT-PCR. Levels of mRNA expression are shown as the fold induction relative to the expression in WT without DSS administration (assigned the value “1”). *P < 0.05 vs WT within each day, ^†^*P* < 0.05 vs non-DSS-treated WT mice, and ^‡^*P* < 0.05 vs non-DSS-treated KO mice; 2-way ANOVA followed by Tukey multiple comparison test (*n* = 7 to 12)

We next examined the effect of mPGES-1 genetic deletion on the population of FoxP3^+^CD25^+^ Tregs in LPMCs isolated from colons with DSS-induced colitis. The population of FoxP3^+^CD25^+^ Tregs was larger and the number of these cells was higher in mPGES-1^−/−^ mice than in WT mice (Fig. 8). Furthermore, mPGES-1^−/−^ mice showed higher expression of colonic IL-10 (an anti-colitis cytokine also produced by Tregs). These data imply that—in addition to its visible anti-colitis activity through regulation of Th17/Th1 immunity during colitis—mPGES-1–driven PGE_2_ may act as an enhancer of colitis by suppressing anti-colitis activity mediated by Tregs.

### Attenuated symptoms of DSS-induced colitis by CD4^+^ T cell depletion in mPGES-1^−/−^ mice

**Fig. 9:**
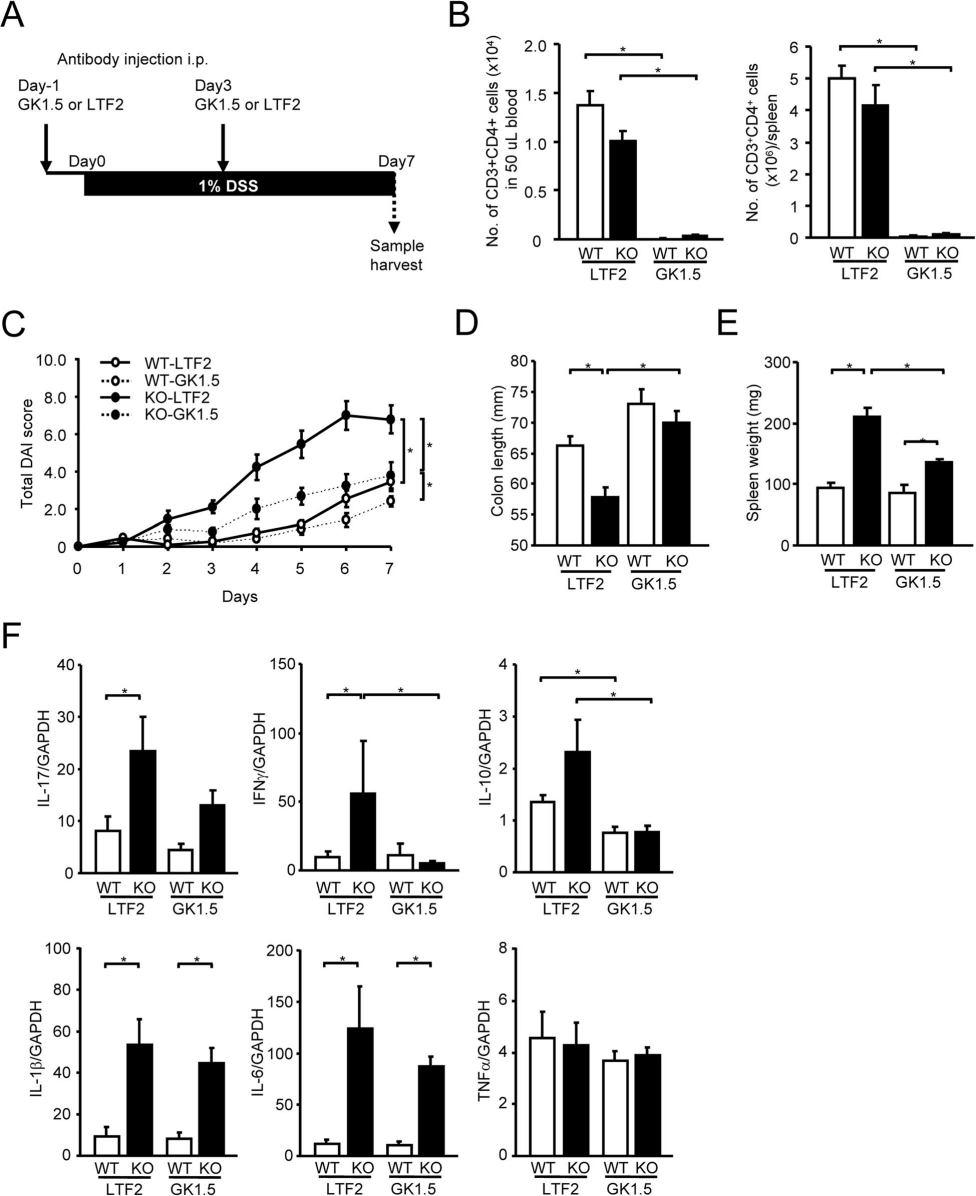
Effect of CD4 positive T cell depletion on the exacerbated DSS-induced colitis in mPGES-1^−/−^ mice. **A** Schematic representation of the experimental plan. **B** The efficacy of *in vivo* CD4^+^ T cell depletion was confirmed by flow cytometry analysis of T cell population in the peripheral blood and spleen (*n* = 9 to 11). **C** Time course of change in total disease activity index (DAI) score of WT and mPGES-1^−/−^ mice after indicated days of exposure to 1% DSS (*n* = 9 to 11). On day 7 after the start of exposure to 1% DSS, the length of the colon (**D**) and weight of the spleen (**E**) were measured as indirect markers of inflammation (*n* = 9 to 11). **F** Expressions of mRNA for IL-17A, IFNγ, IL-10, IL-1β, IL-6, and TNFα in colon from mice treated with 1% DSS for 7 days were analyzed by real-time RT-PCR (*n* = 9 to 11). Levels of mRNA expression are shown as the fold induction relative to the expression in WT mice without DSS administration (assigned the value “1”). *P < 0.05; 2-way ANOVA followed by Tukey multiple comparison test

To investigate the functional role of T cells in the exacerbated DSS-induced colitis under mPGES-1 deficiency, the course of DSS-induced colitis in mPGES-1^−/−^ mice were studied upon CD4^+^ T cell depletion by the treatment with anti-CD4 monoclonal antibody (Clone GK1.5) prior to DSS administration (Fig. 9A). The efficacy of CD4^+^ T cell depletion was confirmed by FCM analysis of T cell population in the peripheral blood, spleen, and LPMCs (Figs. 9B and S2). The treatment with GK-1.5 effectively reduced the number of CD3^+^CD4^+^ T cell *in vivo,* whereas control antibody (clone LTF-2) had no effect. Both GK1.5 and LTF-2 did not affect the population of CD3^+^CD8^+^ T cells and CD11b^+^ cells (data not shown). As shown in Fig. 9C, the total DAI colitis scores were significantly lower in GK1.5-treated mPGES-1^−/−^ mice than in LTF-2-treated mPGES-1^−/−^ mice. The colon shortening and splenomegaly in mPGES-1^−/−^ mice were also significantly improved by the treatment with GK1.5 (Fig. 9C and D). These results suggested the requirement of CD4^+^ T cells in the exacerbation of DSS-induced colitis under mPGES-1 deficiency. Alternatively, even though the colitis and splenomegaly in mPGES-1^−/−^ mice were largely suppressed by the treatment of GK1.5, these were remained to be significantly different when compared to GK1.5-treated WT mice. The level of IL-17 mRNA was 50% lower in GK1.5-treated mPGES-1^−/−^ mice than in LTF-2-treated mPGES-1^−/−^ mice, even though this difference did not reach statistical significance. The levels of IFNγ and IL-10 were significantly lower in GK1.5-treated mPGES-1^−/−^ mice than in LTF-2-treated mPGES-1^−/−^ mice. These results suggest the contribution of CD4^+^ T cells in the upregulation of these cytokines during DSS-induced colitis under mPGES-1 deficiency. On the other hand, the colonic expression levels of IL-1β and IL-6, which were known to be major macrophage cytokines, were significantly higher in mPGES-1^−/−^ mice than in WT mice under either the GK1.5 or LTF2 treatment during DSS-induced colitis. In addition, the colonic expression levels of TNFα did not differ between WT and mPGES-1^−**/−**^ mice after either the GK1.5 or LTF2 treatment.

### Analysis of apoptosis in mPGES-1^−/−^ colons

**Fig. 10:**
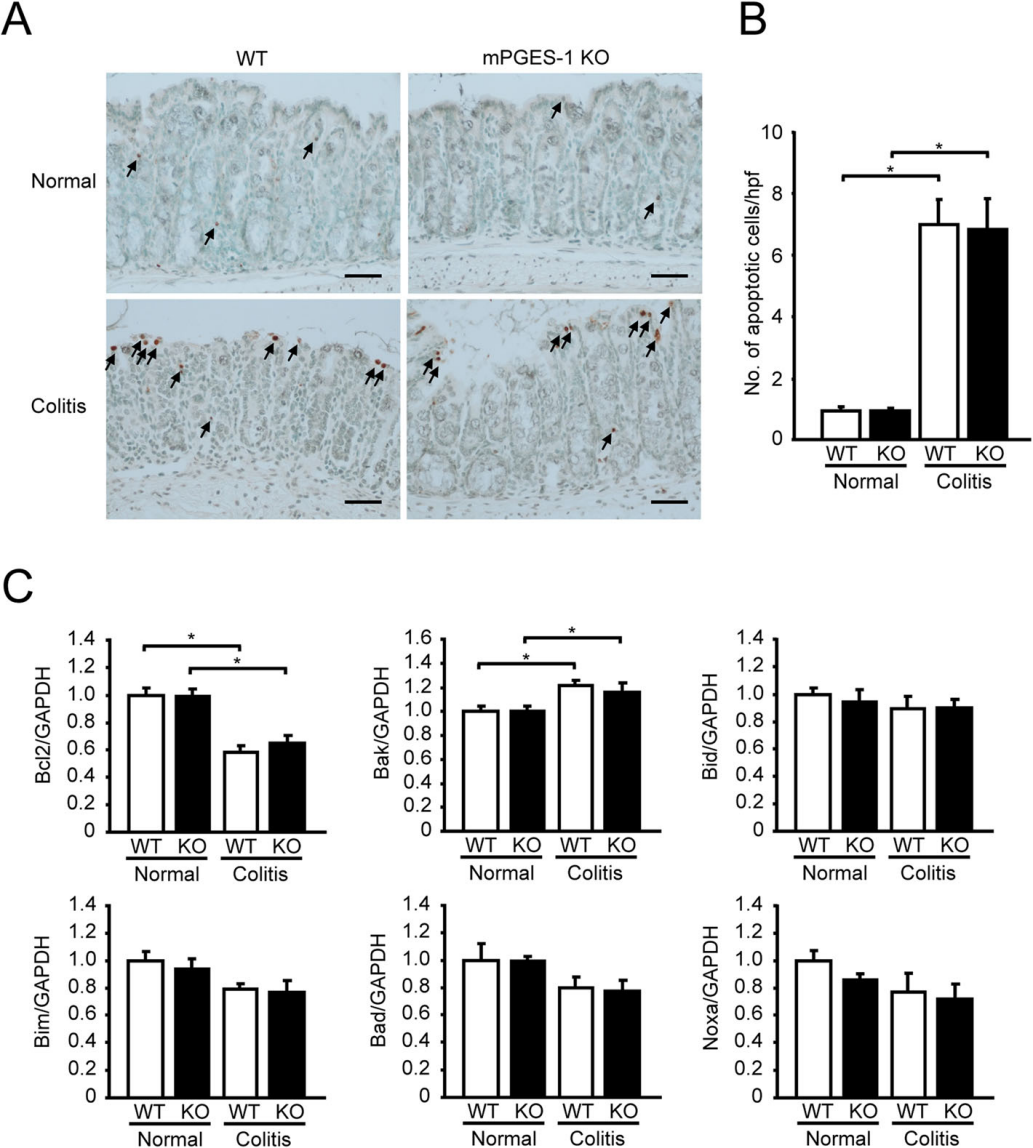
Analysis of apoptosis in mPGES-1^−/−^ colons. **A** Representative images of Swiss-roll colon sections on day 7 after the start of exposure to 1% DSS. Arrows indicate apoptotic cells. Scale bar, 50 μm. **B** Quantification of apoptotic cells in colon (*n* = 5). **C** mRNA expressions of an anti-apoptotic factor Bcl2 and apoptotic markers, Bak, Bid, Bim, Bad, and Noxa, in colon from mice treated with 1% DSS for 7 days were analyzed by real-time RT-PCR (*n* = 7 to 10). Levels of mRNA expression are shown as the fold induction relative to the expression in WT mice without DSS administration (assigned the value “1”). *P < 0.05; 2-way ANOVA followed by Tukey multiple comparison test

*In situ* apoptosis analysis revealed that apoptotic cells were basally detectable in the colon from mPGES-1^−/−^ mice to the same extent as in the colon from WT mice. (Fig. 10A and B). After DSS administration, the number of apoptotic cells significantly increased in both WT and mPGES-1^−/−^ mice. mPGES-1 deficiency did not affect the number of apoptotic cells in the colon not only in colitis but also in the healthy condition. In both WT and mPGES-1^−/−^ colon, mRNA expression of anti-apoptotic factor Bcl2 was significantly decreased by DSS, while mRNA expression of apoptotic marker Bak was significantly increased. These expressions were correlated well with the increased number of apoptotic cells observed after DSS administration in both WT and mPGES-1^−/−^ mice. The colonic expressions of other apoptotic markers, Bid, Bim, Bad, and Noxa, were not changed by DSS treatment in either the WT or mPGES-1^−**/−**^ mice.

## Discussion

The present study demonstrates that mPGES-1 plays an important role in the pathogenesis of DSS-induced colitis by showing that genetic deletion of mPGES-1 resulted in greater inflammatory responses, including multiple colitis parameters and histologically characteristic features of colitis. We also show marked facilitation of colonic Th17 and Th1 immunity under mPGES-1 deficiency. Furthermore, our findings imply the possible potential for mPGES-1 as a pathogenic factor of colitis by suppressing anti-colitis activity mediated by Tregs.

mPGES-1 deficiency enhanced the severity of colitis, with massive weight loss, diarrhea, intestinal bleeding, severe epithelial damage, and infiltration of inflammatory cells after exposure to low-dose DSS, which caused only mild colonic injury in WT mice. A previous study reported that mPGES-1 genetic deletion enhanced colonic ulceration and caused higher mortality during colitis induced by a relatively high dose of DSS [31]. In another report, the fecal blood score was higher in mPGES-1^−/−^ mice than in WT mice when mice were treated with a low molecular weight of high-dose DSS [30]. These studies partly support the findings of our study that mPGES-1 has a protective role during colitis. In a previous study on upstream COX isozymes, mice lacking COX-2 (COX-2^−/−^ mice) were more susceptible to DSS-induced colitis than those lacking COX-1 (COX-1^−/−^ mice); however, after exposure to DSS, COX-1^−/−^ mice were more susceptible to symptoms of colitis than WT mice [52], suggesting that both COX-1 and COX-2 are involved in colon injury. Pharmacological inhibition of both COX-1 and COX-2 and the resultant decrease in the level of intestinal PGE_2_ have also been shown to be responsible for NSAID-dependent exacerbation of DSS-induced colitis [53]. Furthermore, in a study on mice lacking EP receptor subtypes (EP^−/−^ mice), it has been clearly demonstrated that only EP_4_^−/−^ mice developed severe colitis with DSS, while the colitis parameters of EP_1_^−/−^, EP_2_^−/−^, and EP_3_^−/−^ mice were similar to those of control WT mice [13]. Similarly, a number of studies with an EP antagonist/antagonist further support the essential role of PGE_2_ in DSS-induced colitis and that it mediates its effects *via* EP_4_ signaling [13, 54, 55]. Our findings, together with those of earlier studies, suggest that the COX/mPGES-1/PGE_2_ axis could play a pivotal role in DSS-induced colitis predominantly *via* EP_4_ signaling.

One of the important findings of our study is that a lack of mPGES-1 enhanced the colonic induction of COX-2 in response to DSS. In addition, mPGES-1 deficiency altered EP_2_ and EP_3_ expression after DSS administration. These results indicate that mPGES-1 and its associated PGE_2_ regulate the expression of their upstream enzymes and downstream receptors in a feedback loop. We also demonstrated that induction of mPGES-1 is essential for the marked increase of colonic PGE_2_ production during colitis. This finding is consistent with similar findings of the response of other tissues and cell types, such as macrophages, embryo fibroblasts, dendritic cells, and splenocytes, to various inflammatory stimuli [23, 25, 56–58]. Regarding upstream COX, previous studies showed that colonic PGE_2_ production is significantly lower in DSS-treated COX-2^−/−^ mice than in both DSS-treated WT and DSS-treated COX-1^−/−^ mice, indicating the predominant contribution of COX-2 to colonic PGE_2_ synthesis during colitis [52]. Thus, coordinated induction of COX-2 and mPGES-1 would be accompanied by an increase in PGE_2_ levels during severe colitis. Interestingly, the colonic level of PGE_2_ was shown to decrease after treatment with a selective COX-1 inhibitor [53], suggesting that COX-1 also partly contributes to PGE_2_ production during colitis. Additionally, colonic tissues of COX-1^−/−^ mice contain almost undetectable basal levels of PGE_2_, indicating that basal PGE_2_ production is largely dependent on COX-1 in the colon [52]. In the present study, we also saw evidence for a significant reduction in basal PGE_2_ production in the absence of mPGES-1 in the colon. mPGES-1 may be differentially contributing to PGE_2_ production, which might be the result of functional coupling to upstream COX-1.

Noteworthy is that we detected increased production of colonic PGD_2_ in mPGES-1^−/−^ mice, which seemed to be due to shunting of PG precursors down the PGD_2_ synthetic pathway in the absence of mPGES-1. We also detected enhanced colonic COX-2 expression as a result of mPGES-1 deletion. These results suggest that an elevation of COX-2 and the resultant increase in the availability of PGH_2_—as the common substrate for the generation of prostanoid—account for the increased levels of PGD_2_ observed in mPGES-1^−/−^ mice. These results are further supported by our previous study with *mPGES-1*^*−/−*^ dendritic cells, which showed shunting toward PGD_2_ production [58]. Because a number of studies demonstrated the anti-colitis activities of PGD_2_ and its biosynthetic PGD synthases in both experimental colitis and human IBD [16, 46, 59, 60], we propose that the increased PGD_2_ may suppress colitis in the absence of mPGES-1. However, our results showed that lack of mPGES-1 conversely exacerbates colitis and suppresses PGE_2_ production, even though levels of anti-colitis PGD_2_ increase; these findings suggest that during colitis, mPGES-1–driven PGE_2_ plays a greater role than PGD_2_ as an anti-colitis prostanoid.

Our study demonstrated for the first time that mPGES-1 is overexpressed in the colonic mucosal epithelium and infiltrated inflammatory cells in underlying connective tissues and the LP in the colon during DSS-induced colitis. In human IBD including both ulcerative colitis and Crohn’s disease, mPGES-1 was also found to be expressed in the epithelial and inflammatory cells that surround the damaged crypt in the colon during active colitis [28]; this result strongly supports our finding of colonic localization of mPGES-1, which we propose to be responsible for elevated colonic PGE_2_ production during colitis. A number of reports indicated the tissue localization of upstream COX isozymes in mice models of colitis [61, 62], and the pattern in human IBD was found to be similar [16, 63]. The reported localizations of COX isozymes are consistent with the mPGES-1 expression observed in the present study. COX-1 is constitutively expressed in the crypt epithelium and LPMCs in both normal and inflamed colon [61]. In contrast, COX-2 is undetectable in normal colon, but during DSS-induced colitis, it is elevated in epithelial cells in the colon [62] and in a number of LPMCs [61]. Notably, a study demonstrated that COX-2 is required in myeloid cells and endothelial cells for protection against DSS-induced colitis, but not in epithelial cells [64]. mPGES-1 might be differently coupled with COX-1 and COX-2, depending on the expression pattern and its localization in tissues and cell-specific manner. A variety of inflammatory and immune cells may be able to express mPGES-1 within the colon tissues and may be differently responsible for PGE_2_ production. In the present study, we identified CD3^+^ cells and CD11b^+^ cells as the component of inflammatory cells expressing mPGES-1 in the underlying connective tissues and colonic LP. In fact, proinflammatory stimuli-activated macrophages and dendritic cells are able to express mPGES-1, resulting in abundant PGE_2_ production *in vitro* [22, 58]. Activated CD4^+^ T cells also express mPGES-1 and produce PGE_2_ in an mPGES-1–dependent fashion [27]. It has been shown that mPGES-1 is detected with COX-2 to some extent in the colonic lamia propria in T cell–driven colitis induced by adoptive transfer of CD4^+^ effector T cells in mice [65]. Additionally, the study revealed that T cells lacking mPGES-1–dependent PGE_2_ production have reduced colitogenicity, whereas mPGES-1 deficiency in non-lymphoid cells facilitates T cell–driven colitis [65]; the authors interpreted their findings as suggesting that the effects of PGE_2_ derived from T cells differ from those of PGE_2_ derived from other non-lymphoid cells. Further, a previous study investigated DSS-associated colon tumorigenesis in Apc^∆14/+^ mice, a mouse model of familial adenomatous polyposis and reported that mPGES-1 is abundantly expressed in infiltrating cells of colonic ulcerated sites and that some of the mPGES-1–expressing cells were vimentin-positive mesenchymal/fibroblast cells [66]. The group also demonstrated the role of mPGES-1 in the early inflammatory phase of developing colonic carcinogenesis in the Apc^∆14/+^ mice. Future studies in mice with tissue-specific deletion of mPGES-1 may further clarify the intrinsic action of mPGES-1 in distinct cell types in the colon during colitis.

In mice with DSS-induced colitis, we further investigated the role of mPGES-1 in pathogenic immunity in IBD. A previous study reported that mice with DSS-induced colitis show a similar expression profile of cytokines and similar histological changes to those observed in human IBD [67]. DSS treatment induces Th17 cytokines (such as IL-17A) and Th1 cytokines (including IFNγ and IL-2) in the colon, and these cytokines are essential for developing colitis [67–69]. In the present study, we demonstrated for the first time that lack of mPGES-1 facilitates the colonic expression of Th17 and Th1 cytokines in DSS-induced colitis. Our study found a significant increase in colonic expression of IL-17A, as well as IFNγ and IL-2, during colitis in mPGES-1^−**/−**^ mice compared with WT mice. In addition, we showed that the colonic expressions of IL-1β, IL-6, and TGFβ1, which are essential cytokines for Th17 cell expansion and differentiation [47–49], were greater in mPGES-1^−**/−**^ mice. Indeed, we found that IL-17A-producing Th17 cells within colonic LP increase in the absence of mPGES-1, suggesting that during colitis, a mPGES-1–dependent mechanism regulates Th17 differentiation and expansion and also lymphocyte infiltration into the inflammatory sites. The present study also demonstrated an increase in colonic IFNγ-producing Th1 cells in colonic LP and an elevated expression of IL-12p35, a subunit of IL-12 that differentially expands Th1 cells. In addition, we found that L-17^+^IFNγ^+^ double positive cells in colonic LP are increased in mPGES-1^−**/−**^ mice than in WT mice. A previous study showed that IFNγ^+^IL-17^+^coproducing CD4^+^ T cells as a pathogenic Th17 cells are specifically enriched in the inflamed mucosal tissue of IBD patients but not healthy individuals [70].These enhanced colonic Th17 and Th1 immune responses observed in mPGES-1^−**/−**^ mice seems to be associated with the abnormalities consistent with the colitis.

IL-23 is one of the prominent targets of recent biological therapies for the treatment of IBD, as well as several autoimmune diseases. IL-23 has been shown to facilitate the expansion and maintenance of Th17 cells, and the IL-17/IL-23 axis has been shown to be relevant in IBD pathogenesis [50]. A previous study demonstrated that PGE_2_-EP_4_ signaling, in combination with IL-23, regulates Th17 cell expansion *in vitro* [71, 72]. However, the present study found that the colonic expression levels of IL-23 components were similar in both WT and mPGES-1^−**/−**^ mice, suggesting that during colitis facilitation of the Th17 immune response in the absence of mPGES-1 might be independent of IL-23.

Previous studies focused on the expression of IL-1β and TNFα as promising proinflammatory cytokines in the absence of mPGES-1 during colitis [30, 31]. We also confirmed that during colitis, a lack of mPGES-1 facilities increased expression levels of IL-1β, in addition to IL-6. On the other hand, in our study, the differences in the expression levels of TNFα between WT and mPGES-1^−/−^ mice during colitis did not reach statistical significance, a finding apparently in contradiction to published data indicating upregulation of TNFα expression in the absence of mPGES-1 in DSS-induced colitis [30]. Previous reports have documented differences in DSS-induced colitis related to the source of DSS [73, 74]. Therefore, the disparate results obtained from our study and the report [30] could be explained by the fact that they performed their studies with low–molecular-weight DSS, whereas we used high–molecular-weight, colitis-grade DSS.

FoxP3^+^CD25^+^ Tregs have been conclusively shown to suppress mucosal inflammation associated with murine colitis [75–77]. In the present study, during DSS-induced colitis, immune suppressive FoxP3^+^CD25^+^ Tregs within colonic LP were markedly increased in mPGES-1^−/−^ mice compared with WT mice. In addition, mPGES-1^−/−^ mice had a higher expression of colonic IL-10, which is well known as an anti-colitis cytokine produced by Tregs and other immune cells. These results suggest that mPGES-1 may act as a pathogenic factor of colitis by negatively regulating immunosuppression mediated by both Tregs and IL-10. The reason why Tregs increase during severe colitis in the absence of mPGES-1 is unknown, but a similar increase of FoxP3^+^ Tregs was observed at the site of intestinal inflammation in patients with IBD [78, 79], even though the Tregs retained potent suppressive activity [80], implying a possible countereffect of FoxP3^+^ Tregs against excessive abnormal immune responses associated with IBD. To date, autocrine and paracrine effects of PGE_2_ have been shown to be involved in the induction and function of FoxP3^+^CD25^+^ Tregs. FoxP3^+^CD25^+^ Tregs express COX-2 and suppress effector T cells by a PGE_2_-dependent mechanism *in vitro* [81]. However, in a colitis model induced by adoptive transfer of T cells, non-lymphoid mPGES-1–dependent PGE_2_ facilitated the expansion of FoxP3^+^ Tregs and contributed to the resultant suppression of colonic inflammation, whereas CD4^+^ effector T cells expressing mPGES-1 had the potential for developing colitis [65]. In addition, it has been reported that PGE_2_-mediated immunosuppressive mechanisms during colitis were independent on the function of Tregs [82]. Thus, the different roles of mPGES-1–associated PGE_2_ in regulating the differentiation and function of Tregs in human IBD remain elusive.

A previous study reported that DSS-induced colitis can be induced in mice depleted of CD4^+^ helper T cell by the treatment with CD4 monoclonal antibody [83]. Alternatively, another report demonstrated with CD4^+^ T cell depletion that T cells of CD4^+^ but not CD8^+^ phenotype are responsible for the modulation of DSS-induced colitis [84]. In our study, CD4^+^ T cell depletion did not show obvious effect on DSS-induced colitis in WT mice, but it effectively reduced symptoms of colitis as well as colonic expression of Th17 and Th1 cytokines in mPGES-1^−/−^ mice, suggesting the requirement of CD4^+^ T cells in the exacerbation of DSS-induced colitis under mPGES-1 deficiency. Interestingly, even though the CD4^+^ T cell depletion was effective in mPGES-1^−/−^ mice, a part of symptoms were still remained to be significantly different when compared with CD4^+^ T cell–depleted WT mice, suggesting that during colitis, CD4^+^ T cell–independent events also play a role to some extent in the absence of mPGES-1. In the present study, colonic expressions of IL-1β and IL-6, major macrophage-related proinflammatory cytokines relevant to IBD, were still exacerbated in mPGES-1^−/−^ mice under the CD4^+^ T cell depletion during colitis. It was previously reported that PGE_2_-EP_4_ signaling modulates macrophage activation and alters the profile of macrophage cytokines [85, 86], suggesting that macrophages in the colon of mPGES-1^−/−^ mice may be excessively activated. The details of events independent of CD4^+^ T cells remain to be studied.

A recent study has clearly demonstrated that PGE_2_-EP_4_ system plays an important role in maintaining homeostasis in the colon. The study indicated that epithelial-specific deletion of EP_4_ leads to exacerbation of DSS-induced colitis, which is associated with enhancement of apoptosis in colonic epithelial cells [15]. The present study showed that mPGES-1^−/−^ mice exhibited exacerbation of DSS-induced colitis, but mPGES-1 deficiency did not affect the number of apoptotic cells in the colon not only in the healthy condition but also in colitis. In fact, a low level of PGE_2_ was still detectable in the mice without mPGES-1, suggesting that cPGES and other PGES isozymes other than mPGES-1 may contribute to PGE_2_ production to maintain colon homeostasis in colon.

The present study clearly demonstrated that mPGES-1 is the main PGE synthase responsible for intestinal PGE_2_ production and that mPGES-1–associated PGE_2_ plays a protective role in IBD, partly by regulating immune systems associated with CD4^+^ helper T cells. Th17/Th1 immune system of the intestinal tract may be a possible representative to explain the mechanism of immunomodulation by CD4^+^ helper T cells in the absence of mPGES-1. mPGES-1 is a promising candidate for drug development because mPGES-1 inhibition could specifically diminish the elevated PGE_2_ production associated with various autoimmune inflammatory diseases. However, in IBD, the protective effect mediated by mPGES-1–driven PGE_2_ appears to be indispensable for preventing the hyperactivation of the pathogenic T cell immune response and resultant intestinal inflammation. Thus, the present study also provides potentially important information on the possible disadvantageous effect of pharmacological mPGES-1 inhibition in patients with IBD. To investigate the therapeutic efficacy and safety of mPGES-1 inhibitors, future studies need to assess whether mPGES-1 inhibitors can mimic the results observed in mPGES-1^−/−^ mice and how their effects differ from those of traditional COX inhibitors.

## Conclusions

mPGES-1 is the main enzyme responsible for colonic PGE_2_ production, and deficiency of mPGES-1 facilitates the development of colitis by affecting the development of colonic T cell–mediated immunity. mPGES-1 might therefore impact both the intestinal inflammation and T cell–mediated immunity associated with IBD.

## Supplementary Information

**Additional file 1: Table S1.** The number of cell subset in splenocytes from mice treated with or without 1% DSS for 7 days.

**Additional file 2: Figure S1.** Change of water and food uptake in DSS-induced colitis. Weekly water uptake of mPGES-1−/− mice was significantly decreased by 1% DSS administration, while no significant difference was observed in comparison with WT mice. mPGES-1−/− mice showed a trend towards lower uptake than WT mice. A significant decrease in food uptake was observed in mPGES-1−/− mice during DSS administration over 7 days, indicating severe symptoms of colitis in mPGES-1−/− mice. *P < 0.05; 2-way ANOVA followed by Tukey multiple comparison test (n  =  3 to 5). **Figure S2.** In vivo depletion of CD4+ T cells by anti-CD4 (clone GK1.5) monoclonal antibody. (A) Schematic representation of the experimental plan (A). Solid allows indicate time-point (days) at which intraperitoneal injection of the antibody were performed. The efficacy of CD4+ T cell depletion was confirmed by FCM analysis of T cell population in the peripheral blood (B), spleen (C) and LPMCs (D). FCM analysis confirmed that anti-CD4 antibody treatment effectively reduced the number of CD3+CD4+ T cells but not CD3^+^CD8^+^ T cells in the peripheral blood, spleen and LPMCs of the treated mice compared to the control mice. *P < 0.05 vs. control; t test (n  =  3).

## Data Availability

Data generated or analyzed during this study are included in this published article and its supplementary information files.

## Abbreviations

IBD: Inflammatory bowel disease
Th: T-helper
Tregs: Regulatory T cells
PG: Prostaglandin
COX: Cyclooxygenase
PGES: PGE synthase
cPGES: Cytosolic PGES
mPGES-1: Microsomal PGES-1
NSAIDs: Nonsteroidal anti-inflammatory drugs
mPGES-1^−/−^ mice: Mice lacking mPGES-1
DSS: Dextran sodium sulfate
WT: Wild-type
PCR: Polymerase chain reaction
DAI: Disease activity index
HGB: Hemoglobin
HCT: Hematocrit
H&E: Hematoxylin and eosin
FITC: Fluorescein isothiocyanate
GAPDH: Glyceraldehyde 3-phosphate dehydrogenase
LPMCs: Lamina propria mononuclear cells
FCM: Flow cytometry
IFNγ: Interferon-γ
LP: Lamina propria
COX-2^−/−^ mice: Mice lacking COX-2
COX-1^−/−^ mice: Mice lacking COX-1
EP^−/−^ mice: Mice lacking EP receptor subtypes

## References

[1] Ananthakrishnan AN (2015). Epidemiology and risk factors for IBD. Nat Rev Gastroenterol Hepatol..

[2] Guan Q (2019). A comprehensive review and update on the pathogenesis of inflammatory bowel disease. J Immunol Res..

[3] Friedrich M, Pohin M, Powrie F (2019). Cytokine networks in the pathophysiology of inflammatory bowel disease. Immunity..

[4] Lee SH, Kwon JE, Cho ML (2018). Immunological pathogenesis of inflammatory bowel disease. Intest Res..

[5] Kojima F, Kapoor M, Kawai S, Crofford LJ (2006). New insights into eicosanoid biosynthetic pathways: implications for arthritis. Expert Rev Clin Immunol..

[6] Kojima F, Matnani RG, Kawai S, Ushikubi F, Crofford LJ (2011). Potential roles of microsomal prostaglandin E synthase-1 in rheumatoid arthritis. Inflamm Regen..

[7] Jakobsson PJ, Thoren S, Morgenstern R, Samuelsson B (1999). Identification of human prostaglandin E synthase: a microsomal, glutathione-dependent, inducible enzyme, constituting a potential novel drug target. Proc Natl Acad Sci U S A..

[8] Murakami M, Naraba H, Tanioka T, Semmyo N, Nakatani Y, Kojima F, Ikeda T, Fueki M, Ueno A, Oh-ishi S, Kudo I (2000). Regulation of prostaglandin E2 biosynthesis by inducible membrane-associated prostaglandin E2 synthase that acts in concert with cyclooxygenase-2. J Biol Chem..

[9] Murakami M, Nakashima K, Kamei D, Masuda S, Ishikawa Y, Ishii T, Ohmiya Y, Watanabe K, Kudo I (2003). Cellular prostaglandin E2 production by membrane-bound prostaglandin E synthase-2 via both cyclooxygenases-1 and -2. J Biol Chem..

[10] Tanioka T, Nakatani Y, Semmyo N, Murakami M, Kudo I (2000). Molecular identification of cytosolic prostaglandin E2 synthase that is functionally coupled with cyclooxygenase-1 in immediate prostaglandin E2 biosynthesis. J Biol Chem..

[11] Sharon P, Ligumsky M, Rachmilewitz D, Zor U (1978). Role of prostaglandins in ulcerative colitis. Enhanced production during active disease and inhibition by sulfasalazine. Gastroenterology..

[12] Narumiya S, Sugimoto Y, Ushikubi F (1999). Prostanoid receptors: structures, properties, and functions. Physiol Rev..

[13] Kabashima K, Saji T, Murata T, Nagamachi M, Matsuoka T, Segi E, Tsuboi K, Sugimoto Y, Kobayashi T, Miyachi Y, Ichikawa A, Narumiya S (2002). The prostaglandin receptor EP4 suppresses colitis, mucosal damage and CD4 cell activation in the gut. J Clin Invest..

[14] Jiang GL, Nieves A, Im WB, Old DW, Dinh DT, Wheeler L (2007). The prevention of colitis by E prostanoid receptor 4 agonist through enhancement of epithelium survival and regeneration. J Pharmacol Exp Ther..

[15] Matsumoto Y, Nakanishi Y, Yoshioka T, Yamaga Y, Masuda T, Fukunaga Y, Sono M, Yoshikawa T, Nagao M, Araki O, Ogawa S, Goto N, Hiramatsu Y, Breyer RM, Fukuda A, Seno H (2019). Epithelial EP4 plays an essential role in maintaining homeostasis in colon. Sci Rep..

[16] Vong L, Ferraz JG, Panaccione R, Beck PL, Wallace JL (2010). A pro-resolution mediator, prostaglandin D(2), is specifically up-regulated in individuals in long-term remission from ulcerative colitis. Proc Natl Acad Sci U S A..

[17] Korelitz BI (2016). Role of nonsteroidal anti-inflammatory drugs in exacerbation of inflammatory bowel disease. J Clin Gastroenterol..

[18] Engblom D, Saha S, Engstrom L, Westman M, Audoly LP, Jakobsson PJ (2003). Microsomal prostaglandin E synthase-1 is the central switch during immune-induced pyresis. Nat Neurosci..

[19] Saha S, Engstrom L, Mackerlova L, Jakobsson PJ, Blomqvist A (2005). Impaired febrile responses to immune challenge in mice deficient in microsomal prostaglandin E synthase-1. Am J Physiol Regul Integr Comp Physiol..

[20] Inada M, Matsumoto C, Uematsu S, Akira S, Miyaura C (2006). Membrane-bound prostaglandin E synthase-1-mediated prostaglandin E2 production by osteoblast plays a critical role in lipopolysaccharide-induced bone loss associated with inflammation. J Immunol..

[21] Trebino CE, Stock JL, Gibbons CP, Naiman BM, Wachtmann TS, Umland JP, Pandher K, Lapointe JM, Saha S, Roach ML, Carter D, Thomas NA, Durtschi BA, McNeish JD, Hambor JE, Jakobsson PJ, Carty TJ, Perez JR, Audoly LP (2003). Impaired inflammatory and pain responses in mice lacking an inducible prostaglandin E synthase. Proc Natl Acad Sci U S A..

[22] Uematsu S, Matsumoto M, Takeda K, Akira S (2002). Lipopolysaccharide-dependent prostaglandin E(2) production is regulated by the glutathione-dependent prostaglandin E(2) synthase gene induced by the Toll-like receptor 4/MyD88/NF-IL6 pathway. J Immunol..

[23] Kapoor M, Kojima F, Qian M, Yang L, Crofford LJ (2006). Shunting of prostanoid biosynthesis in microsomal prostaglandin E synthase-1 null embryo fibroblasts: regulatory effects on inducible nitric oxide synthase expression and nitrite synthesis. Faseb J..

[24] Kapoor M, Kojima F, Qian M, Yang L, Crofford LJ (2007). Microsomal prostaglandin E synthase-1 deficiency is associated with elevated peroxisome proliferator-activated receptor gamma: regulation by prostaglandin E2 via the phosphatidylinositol 3-kinase and Akt pathway. J Biol Chem..

[25] Kojima F, Kapoor M, Yang L, Fleishaker EL, Ward MR, Monrad SU, Kottangada PC, Pace CQ, Clark JA, Woodward JG, Crofford LJ (2008). Defective generation of a humoral immune response is associated with a reduced incidence and severity of collagen-induced arthritis in microsomal prostaglandin E synthase-1 null mice. J Immunol..

[26] Kojima F, Frolov A, Matnani R, Woodward JG, Crofford LJ (2013). Reduced T cell-dependent humoral immune response in microsomal prostaglandin E synthase-1 null mice is mediated by nonhematopoietic cells. J Immunol..

[27] Maseda D, Johnson EM, Nyhoff LE, Baron B, Kojima F, Wilhelm AJ, Ward MR, Woodward JG, Brand DD, Crofford LJ (2018). mPGES1-dependent prostaglandin E2 (PGE2) controls antigen-specific Th17 and Th1 responses by regulating T autocrine and paracrine PGE2 production. J Immunol..

[28] Subbaramaiah K, Yoshimatsu K, Scherl E, Das KM, Glazier KD, Golijanin D, Soslow RA, Tanabe T, Naraba H, Dannenberg AJ (2004). Microsomal prostaglandin E synthase-1 is overexpressed in inflammatory bowel disease. Evidence for involvement of the transcription factor Egr-1. J Biol Chem..

[29] Chassaing B, Aitken JD, Malleshappa M, Vijay-Kumar M (2014). Dextran sulfate sodium (DSS)-induced colitis in mice. Curr Protoc Immunol..

[30] Hara S, Kamei D, Sasaki Y, Tanemoto A, Nakatani Y, Murakami M (2010). Prostaglandin E synthases: understanding their pathophysiological roles through mouse genetic models. Biochimie..

[31] Montrose DC, Nakanishi M, Murphy RC, Zarini S, McAleer JP, Vella AT (2015). The role of PGE2 in intestinal inflammation and tumorigenesis. Prostaglandins Other Lipid Mediat..

[32] Wirtz S, Popp V, Kindermann M, Gerlach K, Weigmann B, Fichtner-Feigl S, Neurath MF (2017). Chemically induced mouse models of acute and chronic intestinal inflammation. Nat Protoc..

[33] Hamamoto N, Maemura K, Hirata I, Murano M, Sasaki S, Katsu K (1999). Inhibition of dextran sulphate sodium (DSS)-induced colitis in mice by intracolonically administered antibodies against adhesion molecules (endothelial leucocyte adhesion molecule-1 (ELAM-1) or intercellular adhesion molecule-1 (ICAM-1)). Clin Exp Immunol..

[34] Cooper HS, Murthy SN, Shah RS, Sedergran DJ (1993). Clinicopathologic study of dextran sulfate sodium experimental murine colitis. Lab Invest..

[35] Vijay-Kumar M, Sanders CJ, Taylor RT, Kumar A, Aitken JD, Sitaraman SV, Neish AS, Uematsu S, Akira S, Williams IR, Gewirtz AT (2007). Deletion of TLR5 results in spontaneous colitis in mice. J Clin Invest..

[36] Ciceri P, Zhang Y, Shaffer AF, Leahy KM, Woerner MB, Smith WG, Seibert K, Isakson PC (2002). Pharmacology of celecoxib in rat brain after kainate administration. J Pharmacol Exp Ther..

[37] Weigmann B, Tubbe I, Seidel D, Nicolaev A, Becker C, Neurath MF (2007). Isolation and subsequent analysis of murine lamina propria mononuclear cells from colonic tissue. Nat Protoc..

[38] Laky K, Kruisbeek AM. In vivo depletion of T lymphocytes. Curr Protoc Immunol. 2016;113(1). 10.1002/0471142735.im0401s113. 10.1002/0471142735.im0401s113 27038463

[39] Diaz-Granados N, Howe K, Lu J, McKay DM (2000). Dextran sulfate sodium-induced colonic histopathology, but not altered epithelial ion transport, is reduced by inhibition of phosphodiesterase activity. Am J Pathol..

[40] Turner JR (2009). Intestinal mucosal barrier function in health and disease. Nat Rev Immunol..

[41] Chan CB, Abe M, Hashimoto N, Hao C, Williams IR, Liu X, Nakao S, Yamamoto A, Zheng C, Henter JI, Meeths M, Nordenskjold M, Li SY, Hara-Nishimura I, Asano M, Ye K (2009). Mice lacking asparaginyl endopeptidase develop disorders resembling hemophagocytic syndrome. Proc Natl Acad Sci U S A..

[42] Bauer W, Rauner M, Haase M, Kujawski S, Arabanian LS, Habermann I, Hofbauer LC, Ehninger G, Kiani A (2011). Osteomyelosclerosis, anemia and extramedullary hematopoiesis in mice lacking the transcription factor NFATc2. Haematologica..

[43] Spencer RP, Pearson HA (1975). The spleen as a hematological organ. Semin Nucl Med..

[44] Kim CH (2010). Homeostatic and pathogenic extramedullary hematopoiesis. J Blood Med..

[45] Schubert TE, Obermaier F, Ugocsai P, Mannel DN, Echtenacher B, Hofstadter F (2008). Murine models of anaemia of inflammation: extramedullary haematopoiesis represents a species specific difference to human anaemia of inflammation that can be eliminated by splenectomy. Int J Immunopathol Pharmacol..

[46] Ajuebor MN, Singh A, Wallace JL (2000). Cyclooxygenase-2-derived prostaglandin D(2) is an early anti-inflammatory signal in experimental colitis. Am J Physiol Gastrointest Liver Physiol..

[47] Bettelli E, Carrier Y, Gao W, Korn T, Strom TB, Oukka M, Weiner HL, Kuchroo VK (2006). Reciprocal developmental pathways for the generation of pathogenic effector TH17 and regulatory T cells. Nature..

[48] Chung Y, Chang SH, Martinez GJ, Yang XO, Nurieva R, Kang HS, Ma L, Watowich SS, Jetten AM, Tian Q, Dong C (2009). Critical regulation of early Th17 cell differentiation by interleukin-1 signaling. Immunity..

[49] Veldhoen M, Hocking RJ, Atkins CJ, Locksley RM, Stockinger B (2006). TGFbeta in the context of an inflammatory cytokine milieu supports de novo differentiation of IL-17-producing T cells. Immunity..

[50] Wilson NJ, Boniface K, Chan JR, McKenzie BS, Blumenschein WM, Mattson JD (2007). Development, cytokine profile and function of human interleukin 17-producing helper T cells. Nat Immunol..

[51] Hsieh CS, Macatonia SE, Tripp CS, Wolf SF, O'Garra A, Murphy KM (1993). Development of TH1 CD4^+^ T cells through IL-12 produced by Listeria-induced macrophages. Science..

[52] Morteau O, Morham SG, Sellon R, Dieleman LA, Langenbach R, Smithies O, Sartor RB (2000). Impaired mucosal defense to acute colonic injury in mice lacking cyclooxygenase-1 or cyclooxygenase-2. J Clin Invest..

[53] Tanaka K, Suemasu S, Ishihara T, Tasaka Y, Arai Y, Mizushima T (2009). Inhibition of both COX-1 and COX-2 and resulting decrease in the level of prostaglandins E2 is responsible for non-steroidal anti-inflammatory drug (NSAID)-dependent exacerbation of colitis. Eur J Pharmacol..

[54] Sann H, Erichsen J, Hessmann M, Pahl A, Hoffmeyer A (2013). Efficacy of drugs used in the treatment of IBD and combinations thereof in acute DSS-induced colitis in mice. Life Sci..

[55] Watanabe Y, Murata T, Amakawa M, Miyake Y, Handa T, Konishi K, Matsumura Y, Tanaka T, Takeuchi K (2015). KAG-308, a newly-identified EP4-selective agonist shows efficacy for treating ulcerative colitis and can bring about lower risk of colorectal carcinogenesis by oral administration. Eur J Pharmacol..

[56] Trebino CE, Eskra JD, Wachtmann TS, Perez JR, Carty TJ, Audoly LP (2005). Redirection of eicosanoid metabolism in mPGES-1-deficient macrophages. J Biol Chem..

[57] Boulet L, Ouellet M, Bateman KP, Ethier D, Percival MD, Riendeau D, Mancini JA, Méthot N (2004). Deletion of microsomal prostaglandin E2 (PGE2) synthase-1 reduces inducible and basal PGE2 production and alters the gastric prostanoid profile. J Biol Chem..

[58] Monrad SU, Kojima F, Kapoor M, Kuan EL, Sarkar S, Randolph GJ, Crofford LJ (2011). Genetic deletion of mPGES-1 abolishes PGE2 production in murine dendritic cells and alters the cytokine profile, but does not affect maturation or migration. Prostaglandins Leukot Essent Fatty Acids..

[59] Hokari R, Kurihara C, Nagata N, Aritake K, Okada Y, Watanabe C, Komoto S, Nakamura M, Kawaguchi A, Nagao S, Urade Y, Miura S (2011). Increased expression of lipocalin-type-prostaglandin D synthase in ulcerative colitis and exacerbating role in murine colitis. Am J Physiol Gastrointest Liver Physiol..

[60] Iwanaga K, Nakamura T, Maeda S, Aritake K, Hori M, Urade Y, Ozaki H, Murata T (2014). Mast cell-derived prostaglandin D2 inhibits colitis and colitis-associated colon cancer in mice. Cancer Res..

[61] Tessner TG, Cohn SM, Schloemann S, Stenson WF (1998). Prostaglandins prevent decreased epithelial cell proliferation associated with dextran sodium sulfate injury in mice. Gastroenterology..

[62] Fukata M, Chen A, Klepper A, Krishnareddy S, Vamadevan AS, Thomas LS, Xu R, Inoue H, Arditi M, Dannenberg AJ, Abreu MT (2006). Cox-2 is regulated by Toll-like receptor-4 (TLR4) signaling: role in proliferation and apoptosis in the intestine. Gastroenterology..

[63] Singer II, Kawka DW, Schloemann S, Tessner T, Riehl T, Stenson WF (1998). Cyclooxygenase 2 is induced in colonic epithelial cells in inflammatory bowel disease. Gastroenterology..

[64] Oshima M, Herschman HR, Ishikawa TO (2011). Cox-2 deletion in myeloid and endothelial cells, but not in epithelial cells, exacerbates murine colitis. Carcinogenesis..

[65] Maseda D, Banerjee A, Johnson EM, Washington MK, Kim H, Lau KS, Crofford LJ (2018). mPGES-1-mediated production of PGE2 and EP4 receptor sensing regulate T cell colonic inflammation. Front Immunol..

[66] Nakanishi M, Perret C, Meuillet EJ, Rosenberg DW (2015). Non-cell autonomous effects of targeting inducible PGE2 synthesis during inflammation-associated colon carcinogenesis. Carcinogenesis..

[67] Egger B, Bajaj-Elliott M, MacDonald TT, Inglin R, Eysselein VE, Buchler MW (2000). Characterisation of acute murine dextran sodium sulphate colitis: cytokine profile and dose dependency. Digestion..

[68] Ito R, Shin-Ya M, Kishida T, Urano A, Takada R, Sakagami J, Imanishi J, Kita M, Ueda Y, Iwakura Y, Kataoka K, Okanoue T, Mazda O (2006). Interferon-gamma is causatively involved in experimental inflammatory bowel disease in mice. Clin Exp Immunol..

[69] Ito R, Kita M, Shin-Ya M, Kishida T, Urano A, Takada R, Sakagami J, Imanishi J, Iwakura Y, Okanoue T, Yoshikawa T, Kataoka K, Mazda O (2008). Involvement of IL-17A in the pathogenesis of DSS-induced colitis in mice. Biochem Biophys Res Commun..

[70] Globig AM, Hennecke N, Martin B, Seidl M, Ruf G, Hasselblatt P, Thimme R, Bengsch B (2014). Comprehensive intestinal T helper cell profiling reveals specific accumulation of IFN-gamma^+^IL-17^+^coproducing CD4^+^ T cells in active inflammatory bowel disease. Inflamm Bowel Dis..

[71] Barrie A, Khare A, Henkel M, Zhang Y, Barmada MM, Duerr R, Ray A (2011). Prostaglandin E2 and IL-23 plus IL-1beta differentially regulate the Th1/Th17 immune response of human CD161(+) CD4(+) memory T cells. Clin Transl Sci..

[72] Yao C, Sakata D, Esaki Y, Li Y, Matsuoka T, Kuroiwa K, Sugimoto Y, Narumiya S (2009). Prostaglandin E2-EP4 signaling promotes immune inflammation through Th1 cell differentiation and Th17 cell expansion. Nat Med..

[73] Araki Y, Mukaisho K, Sugihara H, Fujiyama Y, Hattori T (2010). Proteus mirabilis sp. intestinal microflora grow in a dextran sulfate sodium-rich environment. Int J Mol Med..

[74] Bamba S, Andoh A, Ban H, Imaeda H, Aomatsu T, Kobori A, Mochizuki Y, Shioya M, Nishimura T, Inatomi O, Sasaki M, Saitoh Y, Tsujikawa T, Araki Y, Fujiyama Y (2012). The severity of dextran sodium sulfate-induced colitis can differ between dextran sodium sulfate preparations of the same molecular weight range. Dig Dis Sci..

[75] Sun X, He S, Lv C, Sun X, Wang J, Zheng W, Wang D (2017). Analysis of murine and human Treg subsets in inflammatory bowel disease. Mol Med Rep..

[76] Sakaguchi S, Sakaguchi N, Asano M, Itoh M, Toda M (1995). Immunologic self-tolerance maintained by activated T cells expressing IL-2 receptor alpha-chains (CD25). Breakdown of a single mechanism of self-tolerance causes various autoimmune diseases. J Immunol..

[77] Mottet C, Uhlig HH, Powrie F (2003). Cutting edge: cure of colitis by CD4+CD25+ regulatory T cells. J Immunol..

[78] Maul J, Loddenkemper C, Mundt P, Berg E, Giese T, Stallmach A, Zeitz M, Duchmann R (2005). Peripheral and intestinal regulatory CD4^+^ CD25(high) T cells in inflammatory bowel disease. Gastroenterology..

[79] Yu QT, Saruta M, Avanesyan A, Fleshner PR, Banham AH, Papadakis KA (2007). Expression and functional characterization of FOXP3^+^ CD4^+^ regulatory T cells in ulcerative colitis. Inflamm Bowel Dis..

[80] Saruta M, Yu QT, Fleshner PR, Mantel PY, Schmidt-Weber CB, Banham AH, Papadakis KA (2007). Characterization of FOXP3^+^CD4^+^ regulatory T cells in Crohn's disease. Clin Immunol..

[81] Mahic M, Yaqub S, Johansson CC, Tasken K, Aandahl EM (2006). FOXP3^+^CD4^+^CD25^+^ adaptive regulatory T cells express cyclooxygenase-2 and suppress effector T cells by a prostaglandin E2-dependent mechanism. J Immunol..

[82] Chinen T, Komai K, Muto G, Morita R, Inoue N, Yoshida H, Sekiya T, Yoshida R, Nakamura K, Takayanagi R, Yoshimura A (2011). Prostaglandin E2 and SOCS1 have a role in intestinal immune tolerance. Nat Commun..

[83] Axelsson LG, Landstrom E, Goldschmidt TJ, Gronberg A, Bylund-Fellenius AC (1996). Dextran sulfate sodium (DSS) induced experimental colitis in immunodeficient mice: effects in CD4(+)-cell depleted, athymic and NK-cell depleted SCID mice. Inflamm Res..

[84] Shintani N, Nakajima T, Okamoto T, Kondo T, Nakamura N, Mayumi T (1998). Involvement of CD4^+^ T cells in the development of dextran sulfate sodium-induced experimental colitis and suppressive effect of IgG on their action. Gen Pharmacol..

[85] Yamane H, Sugimoto Y, Tanaka S, Ichikawa A (2000). Prostaglandin E(2) receptors, EP2 and EP4, differentially modulate TNF-alpha and IL-6 production induced by lipopolysaccharide in mouse peritoneal neutrophils. Biochem Biophys Res Commun..

[86] Akaogi J, Yamada H, Kuroda Y, Nacionales DC, Reeves WH, Satoh M (2004). Prostaglandin E2 receptors EP2 and EP4 are up-regulated in peritoneal macrophages and joints of pristane-treated mice and modulate TNF-alpha and IL-6 production. J Leukoc Biol..

