# Supplementary material for: Facilitation of colonic T cell immune responses is associated with an exacerbation of dextran sodium sulfate–induced colitis in mice lacking microsomal prostaglandin E synthase-1
Source: Inflamm Regen. 2022 Jan 4;42:1. doi: 10.1186/s41232-021-00188-1 (PMC8725565; doi:10.1186/s41232-021-00188-1)
Supplement: Supplementary file 1 — Additional file 1: Table S1. The number of cell subset in splenocytes from mice treated with or without 1% DSS for 7 days. [file 41232_2021_188_MOESM1_ESM.doc]

**Table S1. The number of cell subset in splenocytes from mice treated with or without 1% DSS for 7 days.**

Normal Colitis

Cells WT KO WT KO

CD3^+^CD4^+^ cells 5.2±1.3 4.8±1.3 4.1±0.9 3.2 ±0.5

CD3^+^ cells 9.5±2.2 9.1±2.5 7.9±1.7 6.8±1.3

CD4+ cells 5.4±1.4 5.0±1.3 4.3±0.9 3.3±0.5

CD19^+^ cells 10.2±2.6 11.0±3.5 11.2±3.0 14.2±6.1

CD11c^+^ cells 0.4±0.1 0.5±0.2 0.4±0.1 0.3±0.1

CD11b^+^ cells 2.0±0.5 1.7±0.3 2.3±0.7 1.4±0.4

Gr-1^+^ cells 1.0±0.3 0.8±0.2 1.2±0.4 0.8±0.3

The number of cells (x 10^6^) /spleen. Data + SEM (n=4-5).
