# Supplementary material for: Facilitation of colonic T cell immune responses is associated with an exacerbation of dextran sodium sulfate–induced colitis in mice lacking microsomal prostaglandin E synthase-1
Source: Inflamm Regen. 2022 Jan 4;42:1. doi: 10.1186/s41232-021-00188-1 (PMC8725565; doi:10.1186/s41232-021-00188-1)
Supplement: Supplementary file 2 — Additional file 2: Figure S1. Change of water and food uptake in DSS-induced colitis. Weekly water uptake of mPGES-1−/− mice was significantly decreased by 1% DSS administration, while no significant difference was observed in comparison with WT mice. mPGES-1−/− mice showed a trend towards lower uptake than WT mice. A significant decrease in food uptake was observed in mPGES-1−/− mice during DSS administration over 7 days, indicating severe symptoms of colitis in mPGES-1−/− mice. *P < 0.05; 2-way ANOVA followed by Tukey multiple comparison test (n = 3 to 5). Figure S2. In vivo depletion of CD4+ T cells by anti-CD4 (clone GK1.5) monoclonal antibody. (A) Schematic representation of the experimental plan (A). Solid allows indicate time-point (days) at which intraperitoneal injection of the antibody were performed. The efficacy of CD4+ T cell depletion was confirmed by FCM analysis of T cell population in the peripheral blood (B), spleen (C) and LPMCs (D). FCM analysis confirmed that anti-CD4 antibody treatment effectively reduced the number of CD3+CD4+ T cells but not CD3+CD8+ T cells in the peripheral blood, spleen and LPMCs of the treated mice compared to the control mice. *P < 0.05 vs. control; t test (n = 3). [file 41232_2021_188_MOESM2_ESM.ppt]

## Slide 1
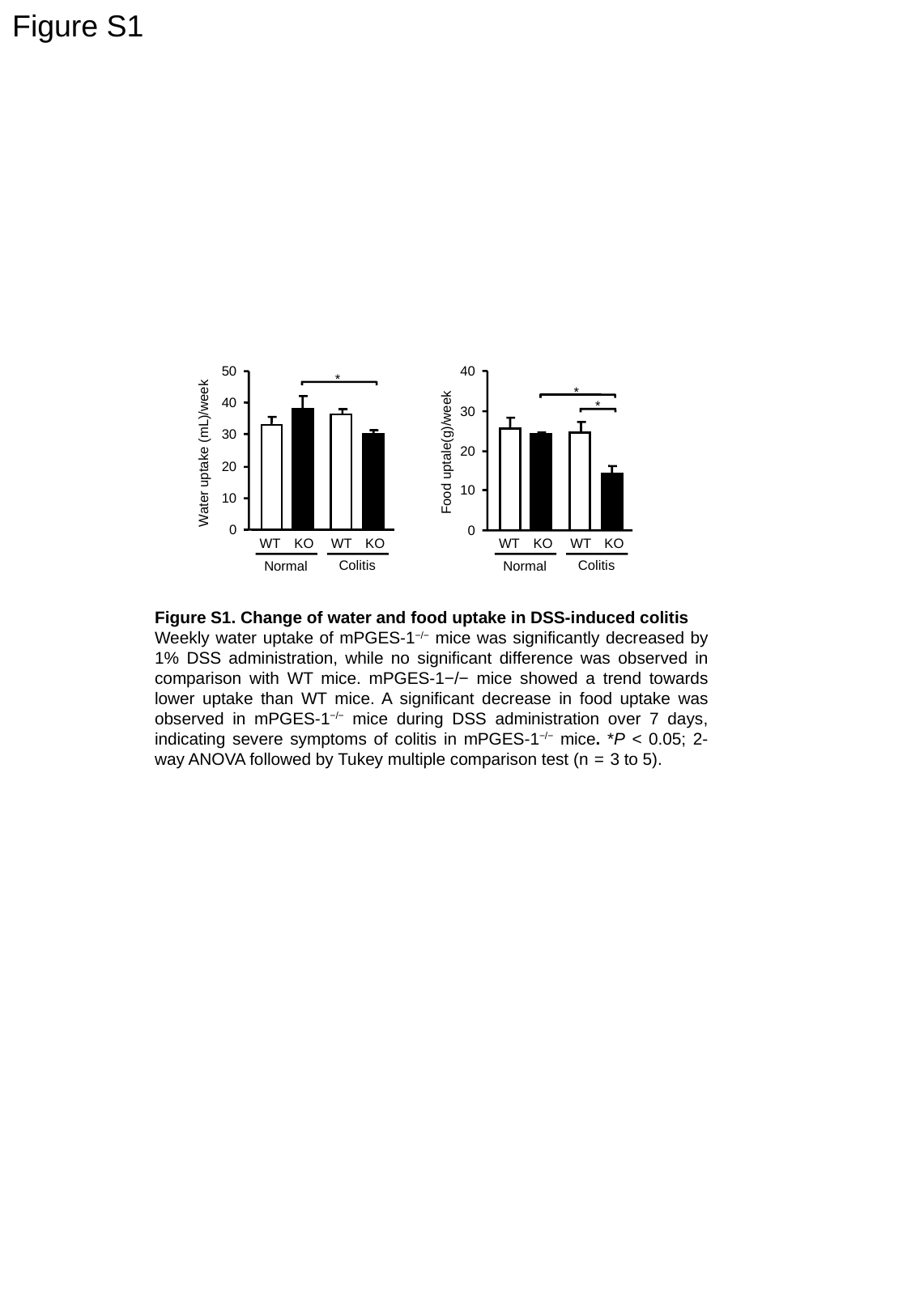

Figure S1
40
50
*
*
*
40
30
30
Food uptale(g)/week
Water uptake (mL)/week
20
20
10
10
0
0
WT
KO
WT
KO
WT
KO
WT
KO
Colitis
Colitis
Normal
Normal
Figure S1. Change of water and food uptake in DSS-induced colitis
Weekly water uptake of mPGES-1−/− mice was significantly decreased by 1% DSS administration, while no significant difference was observed in comparison with WT mice. mPGES-1−/− mice showed a trend towards lower uptake than WT mice. A significant decrease in food uptake was observed in mPGES-1−/− mice during DSS administration over 7 days, indicating severe symptoms of colitis in mPGES-1−/− mice. *P < 0.05; 2-way ANOVA followed by Tukey multiple comparison test (n  =  3 to 5).

## Slide 2
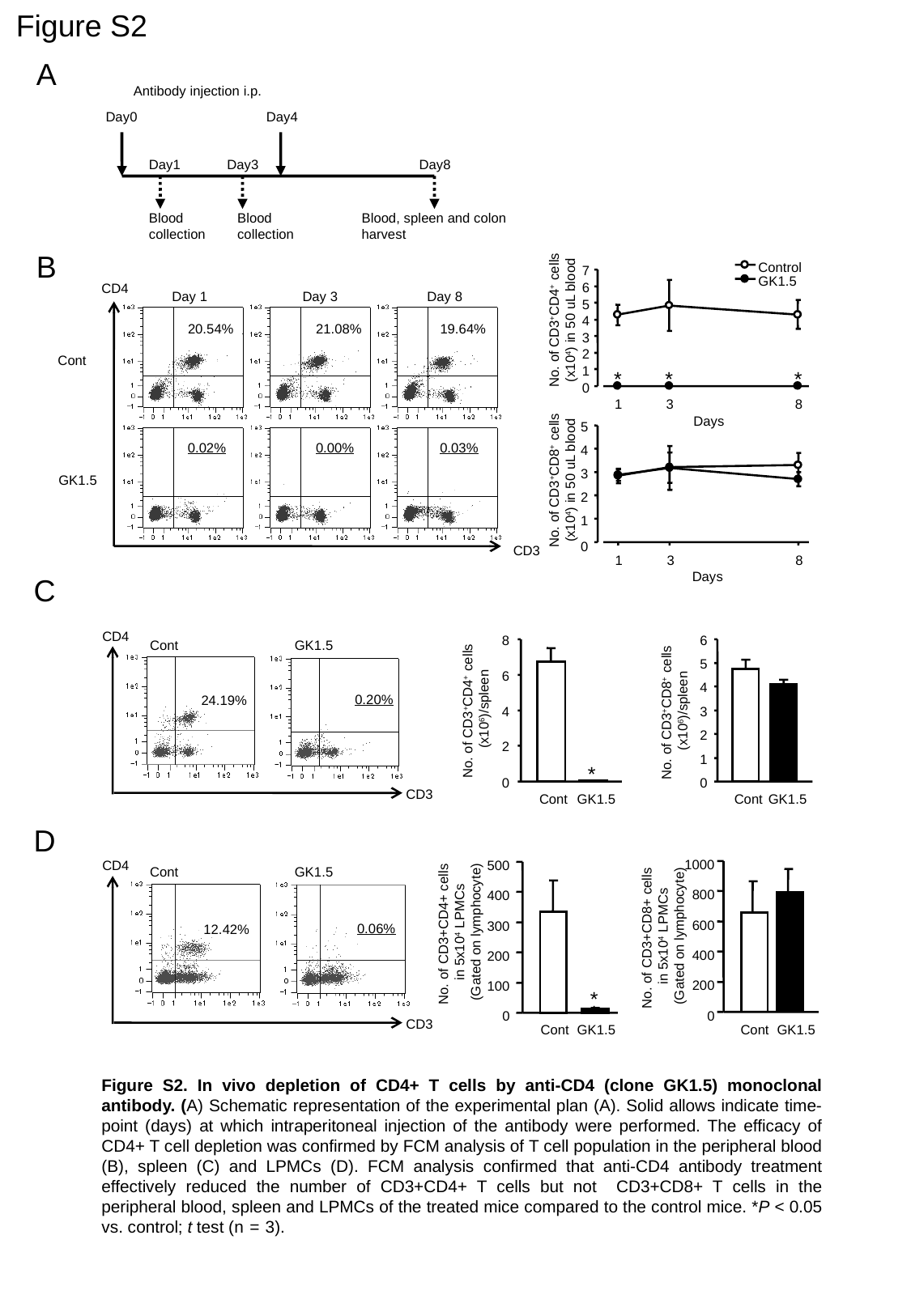

Figure S2
A
Antibody injection i.p.
Day0
Day4
Day1
Day3
Day8
Blood
collection
Blood
collection
Blood, spleen and colon
harvest
Control
7
GK1.5
6
5
No. of CD3+CD4+ cells (x104) in 50 uL blood
4
3
2
*
*
*
1
0
1
3
8
Days
5
4
No. of CD3+CD8+ cells (x104) in 50 uL blood
3
2
1
0
1
3
8
Days
B
CD4
Day 1
Day 3
Day 8
20.54%
21.08%
19.64%
Cont
0.02%
0.00%
0.03%
GK1.5
CD3
C
CD4
Cont
GK1.5
6
8
5
6
4
0.20%
24.19%
No. of CD3+CD8+ cells
(x106)/spleen
No. of CD3+CD4+ cells
 (x106)/spleen
4
3
2
2
1
*
0
0
CD3
Cont
GK1.5
Cont
GK1.5
D
CD4
1000
500
800
400
No. of CD3+CD4+ cells
in 5x10^4^ LPMCs
(Gated on lymphocyte)
No. of CD3+CD8+ cells
in 5x10^4^ LPMCs
(Gated on lymphocyte)
600
300
400
200
200
100
*
*
0
0
Cont
GK1.5
Cont
GK1.5
Cont
GK1.5
0.06%
12.42%
CD3
Figure S2. In vivo depletion of CD4+ T cells by anti-CD4 (clone GK1.5) monoclonal antibody. (A) Schematic representation of the experimental plan (A). Solid allows indicate time-point (days) at which intraperitoneal injection of the antibody were performed. The efficacy of CD4+ T cell depletion was confirmed by FCM analysis of T cell population in the peripheral blood (B), spleen (C) and LPMCs (D). FCM analysis confirmed that anti-CD4 antibody treatment effectively reduced the number of CD3+CD4+ T cells but not CD3+CD8+ T cells in the peripheral blood, spleen and LPMCs of the treated mice compared to the control mice. *P < 0.05 vs. control; t test (n  =  3).
